# Design of synthetic selenopeptides with antioxidant activity for the treatment of XP and non-melanoma skin cancer

**DOI:** 10.3389/fsysb.2026.1686085

**Published:** 2026-04-09

**Authors:** Arthur Alves Coelho, Eduarda Pereira Soares Dias, André Kipnis

**Affiliations:** 1 Laboratory of Immunology of Infectious Diseases, Institute of Tropical Pathology and Public Health, Universidade Federal de Goiás, Goiânia, Brazil; 2 Laboratory of Genetic and Genomic, School of Agronomy, Universidade Federal de Goiás, Goiânia, Brazil; 3 Laboratory of Molecular Bacteriology, Institute of Tropical Pathology and Public Health, Universidade Federal de Goiás, Goiânia, Brazil; 4 Laboratory of Vaccinology, Technological Development Center, Universidade Federal de Pelotas, Pelotas, Brazil

**Keywords:** xeroderma pigmentosum, synthetic biology, peptide design, bioengineering, skin cancer

## Abstract

**Introduction:**

Xeroderma Pigmentosum (XP) is a rare, autosomal recessive disorder characterized by extreme sensitivity to ultraviolet (UV) light. Current treatments primarily focus on symptom management and surgical tumor excisions. Selenopeptides, which possess a modified residue of Cysteine (Selenocysteine), are distinguished for their antioxidant and photoprotective properties. These properties could be beneficial in counteracting the oxidative DNA damage observed in XP lesions.

**Objective:**

Building upon the work initiated by the Brazilian SynBio UFG iGEM Design League team, this study aimed to develop a new approach for designing and expressing synthetic selenopeptides through in silico optimization, to target both XP and non-melanoma skin cancers.

**Methods:**

Five novel sequences of selenopeptides, named Selera, were designed and evaluated through bioinformatic tools. Selera-2 was chosen as the best model designed for its physico-chemical and structural properties and was submitted to docking analysis with therapeutic targets.

**Results and Discussion:**

Docking models of B-RAF and TrxR1 demonstrated to be the most stable binding sites, considering low-binding energy levels and molecular dynamics profile, suggesting possible targets for anti tumor effect. A new recombinant expression plasmid vector was proposed, p-Sec Reg 1, in order to ensure optimal expression for future trials and production. The in silico validation of this innovative approach allows the creation of novel selenopeptides and their prospective applications in the treatment of XP and other skin cancer conditions.

## 1 Introduction

Xeroderma pigmentosum (XP) is a rare autosomal recessive disorder characterized by an extreme sensitivity to ultraviolet light (UV) (Lucero and Horowitz, 2023). This disease results from a failure in nucleotide excision repair (NER), which is responsible for correcting damaged DNA induced by UV radiation, chemical agents, and other environmental factors (Errol et al., 2006). In XP, this failure results in the accumulation of DNA lesions and increased susceptibility to skin malignancies (DiGiovanna and Kraemer, 2012).

XP symptomatology includes severe sunburns after minimal sun exposure, an increased risk of skin cancer development, some neurological issues - including microcephaly, intellectual disabilities, and motor coordination problems (Lehmann et al., 2011) - and ocular problems such as photophobia, keratitis, and conjunctivitis (DiGiovanna and Kraemer, 2012; Kraemer, 1987). The severity of XP symptoms varies depending on specific mutations in the protein genes responsible for DNA repair, which are classified from XP-A to XP-G (Fassihi et al., 2016).

XP incidence varies across different countries, regions, and continents. XP affects one in one million individuals in Europe and the United States (Lehmann et al., 2018). However, the incidence is higher in regions with higher consanguinity rates. For instance, in Japan, the incidence is one in 22,000 (Nishigori and Nakano, 2018), while in Africa it is one in 100,000 individuals (Lehmann et al., 2018). In the countryside of Brazil, Araras-Faina in Goiás state, Brazil, there is a community with the highest incidence of XP in the world: one in 40 individuals is affected by XP (Costa, 2011).

Therapeutic approaches to XP focus on avoiding sun contact, surgical removal of cancerous lesions, laser and photodynamic therapy, and imiquimod use, among other strategies (Hossain et al., 2021). Innovative approaches have also been explored to mitigate XP effects, such as the T4N5 liposomal lotion described by Yarosh et al. (1996), a topical endonuclease cream designed to enhance DNA repair in skin cells. Another promising strategy is gene therapy, a technology aimed at correcting the underlying mutations in XP-related genes. Still, it remains in the early experimental stages (Dupuy and Sarasin, 2015) and has a high cost of development and implementation.

Antioxidants, present in minute quantities in food and the human body, are vital substances that can impede, manage, or halt oxidative processes (Gulcin, 2025). Maintaining redox homeostasis relies on a balance between antioxidants and oxidants such as an imbalance favoring oxidants can lead to oxidative stress. This stress is implicated in the development of numerous diseases, such as cancer (Hecht et al., 2024). Elevated oxidative stress can instigate tumor development and contribute to its progression. This occurs either through direct oxidation of macromolecules or via aberrant redox signaling triggered by oxidative stress (Chaiswing et al., 2018; Urso et al., 2020). Consequently, high levels of reactive oxygen species (ROS) may elevate cancer risk when the body’s antioxidant systems are insufficient to safeguard cells from oxidative stress (Canli et al., 2017). Given the significant role of oxidative stress in carcinogenesis and cancer progression (Hayes et al., 2020), utilizing antioxidants for cancer treatment is a promising approach (Luo et al., 2022).

Some antioxidants have been developed for the treatment of melanoma and XP. Moreno et al. (2019) demonstrated in XP-V cells that UVA-induced oxidative stress contributes to DNA lesions and mutagenesis. Their findings also showed that the antioxidant N-acetylcysteine (NAC) can reverse these deleterious effects *in vitro*, suggesting a plausible protective mechanism. Similarly, Kunisada et al. (2017) reported that NAC alleviated the mutagenic effects of UVA radiation in Simian virus 40 (SV40)-transformed fibroblasts derived from an XP-V patient, further supporting its potential as a protective agent. More recently, Tsujimoto et al. (2023) examined the antioxidant hormone N-acetyl-5-methoxytryptamine (melatonin), and the same group (Tsujimoto et al., 2025) demonstrated both *in vitro* and *in vivo* that melatonin (NPC-15) reduced UV-B-induced cytotoxicity, reactive oxygen species (ROS) production, and UV-induced inflammation in XP-A-deficient mice.

Peptide design for therapeutics is an emerging area for different diseases, such as diabetes, cardiovascular diseases, gastrointestinal diseases, and cancer, among others (Wang et al., 2022). There are several synthetic peptide designs according to treatment strategies, such as Immunogenic Cell Death (ICD) (Aria and Rezaei, 2023). ICD is a regulated cell death process that triggers an immune response against specific targets, such as cancerous cells (Arimoto et al., 2024). This process is characterized by the release of damage-associated molecular patterns (DAMPs) from dying cells, including calreticulin (CRT), high-mobility group box 1 (HMGB1), and extracellular ATP. These DAMPs activate pattern recognition receptors (PRRs) on innate immune cells like dendritic cells and macrophages, leading to their maturation and subsequent activation of tumor-specific T-cell. Other pathways contributing to ICD involve endoplasmic reticulum stress, oxidative stress, and the release of type I interferons (Ahmed and Tait, 2020). For instance, Garg and Agostinis (2014) demonstrated that excessive accumulation of reactive oxygen species (ROS) can cause mitochondrial damage, which then induces ICD. Selenopeptides also have already been described as potential ICD-inducing agents, synergistically acting with chemotherapeutics for enhanced tumor killing activity (Wei et al., 2022).

Selenopeptides are small protein sequences embedded with the unusual amino acid selenocysteine (Sec), a cysteine residue with a Selenium atom (-SeH) instead of the thiol group (-SH) (Hatfield et al., 2014). The mechanism of Sec incorporation is dependent on the selenocysteine incorporation sequence (SECIS) (Serrão and Scortecci, 2020), an mRNA element responsible for forming a hairpin downstream in Bacteria and at the 3′untranslated region (UTR) in Archaea and Eukarya (Su et al., 2005). This hairpin causes a change in UGA codon interpretation and promotes selenocysteine incorporation. This amino acid is often considered a key mechanism in regulating oxidative stress (Ye et al., 2022), similar to the cumulative DNA damage that leads to the symptoms of XP, highlighting the potential of selenopeptides for managing the condition (Hayashi, 2008). Besides the great potential and broad applications, few studies have undergone *in silico* proposal and development of synthetic sequences of selenopeptides, mainly due to the challenges concerning their recombinant expression or chemical synthesis (Wang et al., 2021).

Plasmids are circular pieces of DNA located outside the main chromosome and can copy themselves independently (Godbey, 2014). In the biotechnology field, many of them are designed to express proteins heterologously in microorganisms, such as *Escherichia coli* (Rosano et al., 2019), due to their ease, speed of production, and low cost (İncir and Kaplan, 2024). Specialized vectors are utilized for the heterologous expression of selenopeptides. These vectors are equipped with essential genes and regulatory elements for selenocysteine incorporation, such as those encoding selenoprotein biosynthesis factors (Mukai et al., 2018). For selecysteine recombinant expression, several plasmid designs were proposed (Rengby et al., 2004; Gursinsky et al., 2008; Mukai et al., 2018; Wright and O’Donoghue, 2023), but none of them proved to be suitable and low-cost for high-yield production.

During the year 2023, our group organized the first iGEM team from the State of Goiás, Brazil, and proposed a novel selenopeptide sequence as a bioactive compound for the topical treatment and prophylaxis of XP and non-melanoma skin cancers. The work involved the predictive understanding and manipulation of biological networks involved in selenopeptides recombinant expression, aligning with the broader goal of Systems Biology–to design, model, and control complex biological systems in a holistic and integrative manner. Herein, our objective is to provide, for the first time, an approach for the design and recombinant expression of novel synthetic selenopeptides in *E. coli* through *in silico* optimization of promising antitumoral and antioxidant peptides for the treatment of XP and Non-melanoma skin cancer conditions.

## 2 Methodology

### 2.1 Selection of molecular templates for selenopeptide construction

Template sequences were selected according to their described anti-tumoral activity, particularly those subjected to tests (*in vitro* and/or *in vivo*) with melanoma and non-melanoma cell lines and antioxidant activity, and availability of amino acid sequence, since not all investigated compounds have elucidated structures. The template research was conducted on the open scientific database Pubmed (https://pubmed.ncbi.nlm.nih.gov/), and Cancer PPD 2.0 (Chauhan et al., 2025) (https://webs.iiitd.edu.in/raghava/cancerppd2/).

### 2.2 Design and proposal of novel selenopeptide sequences

Novel selenopeptide sequences were proposed based on the incorporation of selenocysteine and antioxidant standard residues (cationic residues and C-terminal groups) to original sequence templates (Table 1). Mostly, the non-canonical selenocysteine amino acid, represented by letter U, was added to all designed sequences, especially in mid and final C-terminal positions, to raise the rate of successful Sec insertion due to 3′ UTR SECIS proximity. Addition of positively charged residues, such as lysine (K) and histidine (H), was also prioritized in order to contribute to its capability of scavenging highly reactive species, such as UV-induced ROS.

**TABLE 1:** Synthetic selenopeptides sequence design criteri**a**.

Origin	Description
Size	12–35 residues long
Sec insertion	1–4 residues
Sec position	Mid-final chain (C terminal)
Net charge	+1< total net charge < +4
Cationic residues insertion	Arginine (R), lysine (K) and hystidine (H)

The tridimensional structural models were generated by online software AlphaFold2 (https://alphafold.ebi.ac.uk/) and I-Tasser (Zheng et al., 2021) (https://zhanggroup.org/I-TASSER/), and the quality of the structures assessed was taken into consideration for the following tests. Multiple sequence alignment was conducted on ClustalOmega (https://www.ebi.ac.uk/jdispatcher/msa), and structural alignments were obtained from Pymol (https://www.pymol.org/). The prediction of theoretical physicochemical properties was assessed through the online software Pepdraw (https://pepdraw.com/).

### 2.3 Selection of molecular targets for XP non-melanoma skin cancer treatment and prophylaxis

The selection of candidate targets for the synthetic peptide’s inhibitory activity considered the assessment of ongoing clinical research information and data on treatment efficacy from promising therapeutic strategies. All chosen targets presented one or more roles on metabolic pathways involved in tumor proliferation and survival, and were subsequently filtered according to the quality of their three-dimensional structure model and its functional description available at the PDB database (https://www.rcsb.org/).

### 2.4 Molecular docking and molecular dynamics of synthetic sequences and targets

To elucidate possible ligand-receptor binding interaction, molecular docking was conducted using the model of Piper FFT-based rigid docking through a clustering program provided by ClusPro 2.0 (https://cluspro.org/) and coarse-grained protein model of flexible docking through CABS-dock server (https://biocomp.chem.uw.edu.pl/CABSdock/). This tool simulates billions of possible ways that a ligand (peptide) could bind to a target (protein receptor), and then filter according to most stable clusters (lowest-energy structures), a high number of cluster members for a particular predicted binding site is extremely useful because it provides a strong indication that this predicted site is the most likely and stable location where the peptide actually binds to the protein (Kozakov et al., 2017). Therefore, the best models were ranked according to cluster size and were structurally evaluated using the software Pymol (https://pymol.org/) and PDBsum Generate (https://www.ebi.ac.uk/thornton-srv/databases/pdbsum/).

Molecular dynamics analysis was also conducted to better understand the molecular stability conformation of the models that were generated. The analysis was conducted using the open source program GROMACS (https://ftp.gromacs.org/gromacs/gromacs-2025.3.tar.gz), and the commands script available trough MD tutorials (http://www.mdtutorials.com/gmx/lysozyme/index.html) (Lemkul, 2019). Overall, the SPC water model was employed to simulate water molecules within the OPLS-AA/L force field. The system was neutralized according to the total net charge of each model, and 0.15 M of chloride and sodium ions was added and randomly distributed within the solvation system. After constructing the solvation system, energy minimization was performed, and the system was stabilized through isothermal-isobaric (NPT) equilibration, at 300 K and pressure at 1 atm. Following this setup, 10 ns of NPT simulations were run, with trajectories saved at 10 ps intervals.

### 2.5 Plasmid construction for selenopeptide recombinant expression

The plasmid sequence was optimized to serve as a functional vector to express the selenocysteine protein along with its secondary factors, including SelA, SelB and tRNA sec genes. Because the SECIS region was already present in the Selera-2 CDS, it was not necessary to include it in our plasmid. All the plasmid sequence design and manipulations were carried out using the Benchling platform (https://www.benchling.com/). The codon usage optimization virtual tool was employed to enhance protein expression in *E. coli*.

Initially, the process involved searching for standardized biological parts (Biobricks) in publicly available databases such as GenBank (https://www.ncbi.nlm.nih.gov/genbank/) and the iGEM Parts Registry (https://parts.igem.org/Main_Page). Biobricks are standard DNA sequences that flank genetic elements, enabling their assembly into larger modules with defined functions. The standardization offered by Biobricks allows for a reduction in variability in the construction of new genetic circuits, facilitating protocols for assembling new genetic circuits (Dress et al., 2011). The sequences of all BioBricks used in this study are provided in Supplementary Material S1. Benchling’s *in silico* tools were used to cleave the pSB1C3 plasmid backbone at the NotI restriction site, which facilitated downstream cloning via the Assembly Wizard. The pSB1C3 plasmid was selected to compose pSec-Reg because it is a high-copy-number BioBrick backbone (100–300 copies per cell). It also confers chloramphenicol resistance and is the standard cloning backbone utilized by the iGEM Registry (Che, 2008).

Separately, the inserts containing essential genes for Selera-2 expression (Insert 1) and the target peptide gene (Selera-2) (Insert 2) were constructed. Biobrick prefix (5′-GAA​TTC​GCG​GCC​GCT​TCT​AG-3′) and suffix (5′-TAC​TAG​TAG​CGG​CCG​CTG​CAG-3′), available on https://www.addgene.org/synthetic-biology/guide/, were added to the termini of the inserts one and 2, respectively, to ensure compatibility with the Biobrick assembly standard. The first insert contains (1) promoter lac, (2) ribosome binding site (RBS), (3) SelA gene, (4) SelB gene, (5) tRNA sec gene, and (6) rpoC Terminator. The second insert functions under T7 promoter activity, presenting a RBS sequence, Selera-2, and a T7 terminator in that order. We used Benchling’s “Assemble sequences and oligos by concatenation” tool to merge these elements without relying on cohesive ends. After assembly, the insert was virtually digested with Not I, which cuts only within the BioBrick prefix and suffix, thereby generating cohesive ends only at those standard regions. The pSec-Reg expression vector was constructed by ligating the pSB1C3 backbone and the insert. Both were virtually digested with the Not I restriction enzyme, and the ligation was performed using the “Assemble DNA by digest and ligate” tool (Supplementary Material S1). Maps of insert 1, insert 2, and plasmid pSec-Reg were visualized using SnapGene software (https://www.snapgene.com/).

## 3 Results

### 3.1 Template selection and novel sequences design

After filtering and analyzing validated short peptides described in literature, we narrowed our template selection to five molecules with different mechanisms or cellular interfering pathways each, as shown in Table 2. YR-10 is a derived yeast extract purified peptide with relevant antioxidant activity correlated to its structural composition, such as Tyr, Arg, and His residues. Recent research demonstrated that its capacity to induce oxidative stress protective activity is correlated to the amino acid composition, showing an important structural baseline for antioxidant-derived specimens (Mirzaei et al., 2019).

**TABLE 2:** Natural, isolated and synthetic antioxidants and anti-melanoma peptides.

Name	Origin	Described activity	References
YR-10	Yeast extract	A short peptide sequence was identified and purified from the yeast extract of *Saccharomyces cerevisiae*	Mirzaei et al. (2015)
Rb4	Human transmembrane lipoprotein	Derivative peptide sequence from PLP-2, a small transmembrane lipoprotein, with an important role in cancer cells' survival and proliferation	Maia et al. (2022)
LW2	Bovine serum albumine site IIA	A small synthetic peptide, derived from a sequence of bovine serum albumin site IIA, that exerts optimal oxidative protection from ultraviolet irradiation	Wu et al. (2021)
LTX-315	Bovine lactoferricin (LfcinB)	A cationic antimicrobial peptide capable of inducing the immunogenic cell death of tumor cells	Camilio et al. (2014)
OS-LL11	*Odorrana schmackeri* (amphibian)	An amphibian-derived peptide with anti-photodamage properties	Xie et al. (2021)

Rb4 is a synthetic peptide derived from a human lipoprotein, PLP-2, mostly found on endoplasmic reticulum, and commonly upregulated on tumor cells such as gliomas and melanomas (Breitwieser et al., 1997). During an *in vitro* trial with murine melanoma B16F10-Nex2 cells, Rb4 demonstrated high capability of tumor cell death induction, suggesting it as a valuable template for novel peptide derivations (Maia et al., 2022). LW2 is a linear peptide derived from Bovine Serum Albumine Site IIA, that possesses great affinity to phenolic acids. Under UVA irradiation experiment, the peptide LW2 showed protective ability against highly oxidant molecules, elucidating important features conferred by cationic and hydrophobic residues and its capability of acting as a synergistic agent for UV protection (Wu et al., 2021).

LTX-315 is also a bovine-derived peptide, from Bovine Lactoferricin B, that was able to promote a complete regression of B16 melanoma cells in murine models (Camilio et al., 2014). Its mechanism of action is based on cell and mitochondrial membrane disturbance and release of danger-associated patterns that elicit immune system reactivity against tumor cells and can be successfully combined with chemotherapeutics for synergistic activity (Camilio et al., 2019). OS-LL11 is a natural peptide isolated from the amphibian *Odorrana schmackeri*, and was able to scavenge free radicals from mouse skin exposed to UV-B radiation, upregulating substances like superoxide dismutase and glutathione (Xie et al., 2021).

Five novel sequences, all named Selera, were generated, of which two presented more than 30 amino acid residues, while the others were less than 20 residues long (Table 3). Selera-1 also has two protein fusion linkers (GGGGS), necessary to accommodate all three core domains (YGUKHHVCAVR). In comparison to all the designed sequences, Selera-3 presented the highest total net charge (+4), while all of them still possessed negatively charged or apolar residues, and total net charges greater than +1.

**TABLE 3:** Novel synthetic selenopeptide designed sequences.

Name	Origin	Size	Sequence^a^
YR-10	Original	10	YGKPVAVPAR
Selera-1	Modified	43	YGUKHHVCAVRGGGGSYGUKHHVCAVRGGGGSYGUKHHVUAVR
Rb4	Original	24	MADSERLSAPGCWAACTNFSRTRK
Selera-2	Modified	31	MADUSERLHHSAHKKGUWAACTNFSRCTRKU
LW2	Original	11	QNKRFYFRKNQ
Selera-3	Modified	16	QNKKRFHYFRKKUNQU
LTX-315	Original	9	KKWWKKW-dip-K
Selera-4	Modified	15	QRAUKKILWWKKUWU
OS-LL11	Original	11	LLPPWLCPRNK
Selera-5	Modified	15	LLPPWKKLCPRUNUK

^a^ One-letter code amino acids. U = selenocysteine.

**FIGURE 1:**
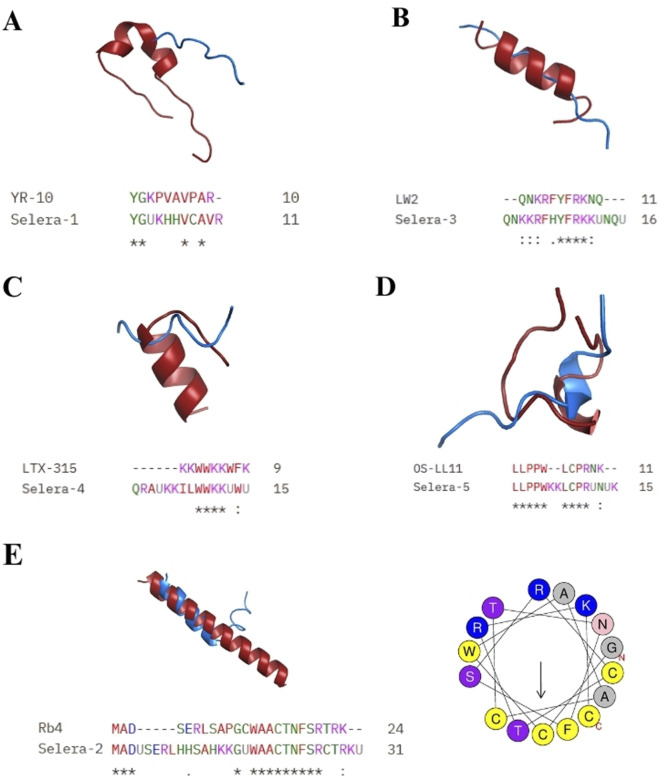
Alignment of predicted structures from template peptides (in blue) and selenopeptides (in red), and sequence comparisons through pairwise alignment (Clustal standard color code) (*) position with conserved residues (:) position of semiconservative residues (.) position of weakly conserved residues. **(A)** YR-10 and Selera-1; **(B)** LW-2 and Selera-3; **(C)** LTX-315 and Selera-4; **(D)** OS-LL11 and Selera-5; **(E)** Rb4 and Selera-2 alignment on the left, and on the right, its helical wheel representation of residues at positions 16–31.

Three-dimensional secondary models were generated to analyse conformational changes, whether detrimental or optimal, due to sequence modifications. Most of the designed selenopeptides exhibited alpha-helix structures (Figures 1A–E **)**, except for Selera-5, which did not present secondary structure but it reproduced the conformation of template peptide OS-LL11 (Figure 1D). Multiple alignment between templates and selenopeptide sequences enabled the visualization of conserved and altered residues, in correspondence to their physicochemical properties (ClustalOmega standard color code). As shown in Figure 1E, Selera-2 was the longest alpha-helix structure obtained, and through the helical wheel representation (positions 16–31), it is possible to observe its amphipathic residue conformation, which is distinguished by a hydrophobic/non-polar domain (indicated by the hydrophobic moment arrow) and a hydrophilic domain composed of polar/charged residues (Gautier et al., 2008).

The physicochemical theoretical properties of the synthetic peptides were also predicted and are shown in Table 4. All peptides presented positive total net charge, as expected, hence potentiating their interaction with nucleophilic species, but also different hydrophobicity according to their amino acid composition. Since the Selera-2 sequence demonstrated to exert alpha-helix conformation through most of its extension, a desirable amphipathic profile, solubility in polar solvents (hydrophobicity greater than +40 Kcal * mol −1), and positive total net charge ≤ +4 at pH 5,5 (median skin pH) it was chosen as the representative molecule for the selenopeptide sequences proposed here, and to proceed to the next functional evaluations.

**TABLE 4:** Physicochemical theoretical properties of synthetic peptides.

Parameters	Selera-1	Selera-2	Selera-3	Selera-4	Selera-5
Molecular weight	4,600.79	3,680.39	2,222.95	2,122.85	1893.84
Net charge (pH 7)	+3	+1	+4	+2	+2
Net charge (pH 5,5)	+8	+4	+5	+3	+3
Isoelectric point (pI)	8.81	8.24	10.64	10.12	9.83
Hydrophobicity (Kcal * mol^-1^)	+58.65	+45.71	+30.56	+23.14	+19.92

#### 3.1.1 Target selection and molecular docking

Four molecular targets, both transmembrane or cytosolic proteins, were chosen for investigation of interaction with Selera-2, as shown in Table 5. More specifically, the first target (B-RAF) is an isoform of the RAF protein, a family of serine/threonine protein kinases that are part of cell proliferation and survival signaling pathways. B-RAF mutations are widely present in melanoma cancerous cells (Gray-Schopfer et al., 2005), and have been prospected as therapeutic targets, including for non-melanoma cancers (Hyman et al., 2015). Therefore, B-RAF was included as a target in this work in its wild-type form for prospective affinity with conservative/semiconservative sites.

**TABLE 5:** Selection of molecular therapeutic targets for non-melanoma skin cancer.

Target	Origin	PDB ID	Cell site
B-RAF	RAF family (protein kinases)	1UWH	Cytosolic
EGFR	Human epithelial growth factor	1IVO	Transmembrane
SMO	Human smoothened protein	4JKV	Transmembrane
TrxR1	Mammalian thioredoxin reductase	2CFY	Cytosolic

Epithelial Growth Factor (EGFR) is a transmembrane protein that regulates cell growth through tyrosine kinase-mediated signals, and its UV-induced mutation is often associated with tumor cell proliferation in squamous cell skin carcinomas (Spallone et al., 2011). New therapeutics focusing on targeting EGFR are promising alternatives to tackle non-melanoma skin cancer (Rubió et al., 2009). Smoothened Protein (SMO), another transmembrane protein, plays a crucial role in the Hedgehog (Hh) pathway signaling, which in basal-cell carcinomas (BCC) can induce unrestrained proliferation of basal cells of the skin (Spallone et al., 2011). In healthy cells, SMO is suppressed by unbound PITCH proteins, and it contributes to the blockade of Hh target genes cascade signaling; therefore, its inhibition has been proposed as a novel alternative for BCC therapy (Von Hoff et al., 2009).

Thioredoxin reductases (TrxR) are essential enzymes for cellular oxidative stress protection, but the overproduction of TrxR in cancerous cells is associated with their proliferation and prolonged survival (Gencheva and Arnér, 2022). TrxR1 inhibitors have shown promising antitumor effects by inducing oxidative cell death while synergizing with other chemotherapeutics (Hsieh et al., 2024). All the sequences already had the tertiary structure empirically elucidated and available online at the Protein Data Bank.

**FIGURE 2:**
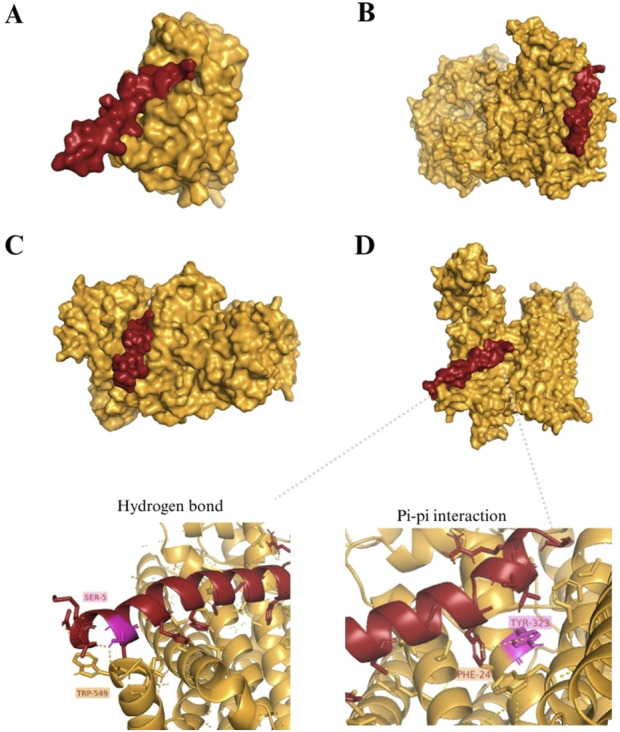
Tridimensional visualization of best docked models from Cluspro 2.0 (highest cluster members), highlighting the molecular surface area of the target (in goldenrod color) and peptide Selera-2 (in red color). Targets: **(A)** B-Raf2; **(B)** EGFR; **(C)** TrxR1; **(D)** SMO docking model and specific protein-peptide interactions.

The docking models with the highest number of cluster members/cluster density, from either Cluspro 2.0 and CABS-dock, can inform most probable conformational models (Table 6). Combining with the observation of the lowest hydrophobic-favored docking energy, which demonstrates great potential for stable interaction by non-molecular bonding, and average ligand RMSD <3 Å as a high-quality prediction (Kurcinski et al., 2015), both B-RAF and TrxR1 were revealed to be more likely stable targets for Selera-2 interaction. The visualization of surface area from best-ranked docked models (Figure 2) allowed the spatial observation of Selera-2’s most probable docking sites, and in depth, Figure 2D demonstrates the interactions between protein-ligand, such as Pi-pi non-bonded contact and hydrogen covalent bond.

**TABLE 6:** Evaluation of peptide-protein target affinity through rigid and flexible docking.

Parameters		B-RAF	EGFR	SMO	TrxR1
ClusPro 2.0					
Highest n° cluster members^a^		159	97	181	398
Electrostatic-favored	Center	−871.3	−876.8	−1,355.7	−833.7
	Lowest energy	−902.9	−1,014.5	−1,355.7	−952.4
Hydrophobic-favored	Center	−1,277.4	−1,107.4	−1835.7	−1,123.9
	Lowest energy	−1,343.0	−1,171.1	−2,209.7	−1,396.3
VdW-favored	Center	−187.2	−193.0	−256.9	−213.7
	Lowest energy	−187.2	−232.1	−274.2	−240.2
CABS-dock					
Cluster density		48.68	26.38	12.9	29.7
Average ligand RMSD (Å)		2.01	7.58	9.6	3.15

^a^ Cluster members from the hydrophobic favored models.

**FIGURE 3:**
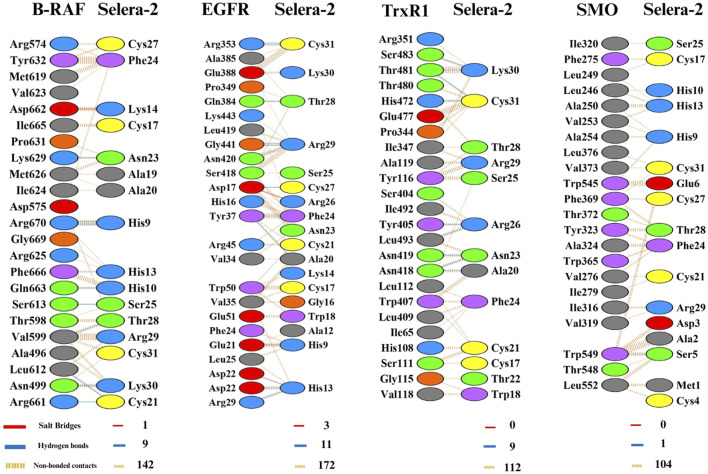
Structural analysis of psycho-chemical interactions between docking models of Selera-2, provided by PDBsum platform. The number of H-bond or Salt bridges lines between any two residues indicates the number of potential bonds between them. For non-bonded contacts, which can be plentiful, the width of the striped line is proportional to the number of atomic contacts. Residues legend colors as disposed by PDBsum, are as it follows: in blue positive residues (His, Lys, Arg), in red negative residues (Asp, GLu), in green neutral residues (Ser,Thr, Asn, Gln), in grey aliphatic residues (Ala,Val, Leu, Ile, Met), in purple aromatic residues (Phe,Tyr, Trp), in orange proline and glycine (Pro, Gly), in yellow cysteine (Cys).

The type of psycho-chemical contacts and residues involved in those interactions was analysed for the four docking models (Figure 3). All models presented non-bonded contacts and hydrogen bond interactions, with the presence of salt-bridges for both B-RAF and EGFR models. Although the PDB sum tool does not display Sec residues with their conventional three-letter code, their presence in the peptide sequence corresponds to the positions Cys4, Cys17, and Cys31, and it is possible to observe that in all models the residues Cys17 and Cys31 were involved in promoting non-bonded interactions and hydrogen bond (Cys31). Phenylalanine (Phe24), an aromatic residue, was also involved in non-bonded contact interactions in all models, and a pi-pi interaction between residues Tyr323 and Phe24, in SMO and Selera-2 models.

**FIGURE 4:**
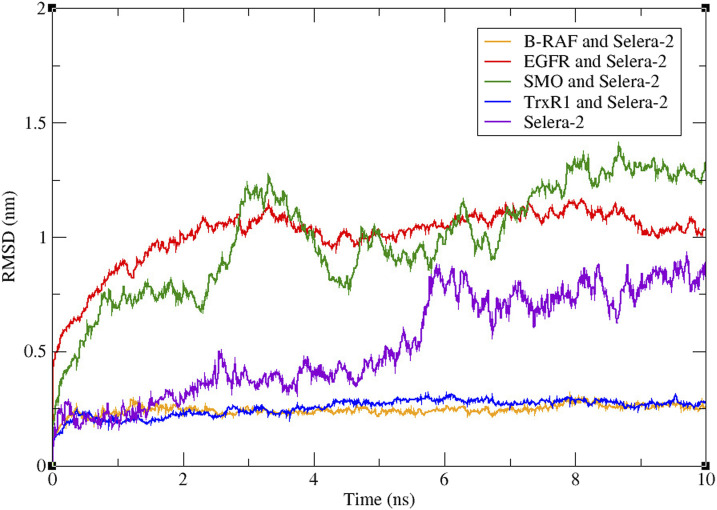
Molecular dynamics simulation of docking models and Selera-2 peptide, in axis y the root mean square deviation (RMSD) in angstroms (Å) and in axis the time of simulation in nanoseconds (ns). In orange there is B-RAF and Selera-2, in red EGFR and Selera-2, in green SMO and Selera-2, in blue TrxR1 and Selera-2, in purple Selera-2.

Molecular dynamic simulation allows observations on complex structural stability under controlled conditions and over time, as shown in Figure 4. The models of B-RAF and TrxR1 demonstrate the most probable stable binding sites, with average RMSD values of 0.245 Å and 0.254 Å, respectively. Although the system equilibrium was not achieved during the timeframe of our simulation for both SMO and EGFR models, it was possible to observe higher average RMSD values for each model, 0.99 Å and 1.00 Å, respectively. Selera-2 was also analysed alone, to observe the difference in molecule behavior under simulation before and after docking, and the average of RMSD obtained for the peptide alone was 0.53 Å. Figure 4. Molecular dynamics simulation of docking models and Selera-2 peptide, in axis y the root mean square deviation (RMSD) in angstroms (Å) and in axis the time of simulation in nanoseconds (ns). In orange there is B-RAF and Selera-2, in red EGFR and Selera-2, in green SMO and Selera-2, in blue TrxR1 and Selera-2, in purple Selera-2.

#### 3.1.2 Optimal recombinant selenopeptide plasmid

**FIGURE 5:**
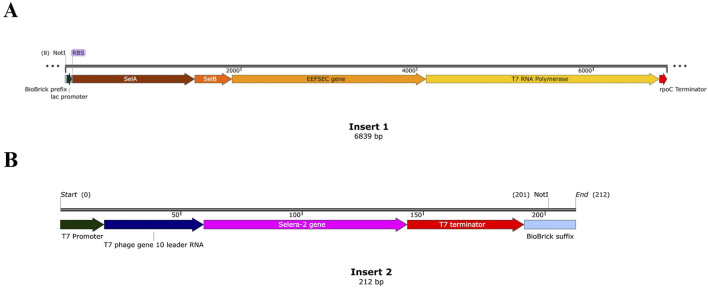
Schematic representation of the two synthetic DNA inserts used in this study **(A)** Insert 1 (6,839 bp) contains the Biobrick prefix (light blue), a lac promoter (dark green) and ribosome binding site at the 5′end (dark blue), followed by the SelA gene (brown), the SelB gene (dark orange), the EEFSEC gene (light orange), and the T7 RNA polymerase gene (yellow). A transcription terminator is located at the 3′end (red) **(B)** Insert 2 (212 bp) includes a T7 promoter (dark green), a RBS - T7 phage gene 10 leader RNA - (dark blue), the synthetic Selera-2 sequence (magenta), a T7 terminator (red) and the Biobrick suffix (light blue). These maps were visualized using SnapGene software.

For the expression of this selenopeptide, a plasmid framework called pSec-Reg (9,078 bp) was proposed (Figure 5 and Supplementary Material S2). The pSB1C3 vector was used as a backbone with two additional promoters added (p*lac* and pT7). The *selA*, *selB,* tRNA sec genes, and T7 RNA polymerase are under the control of the regulated p*lac* promoter (Figure 5A). Additionally, in the presence of T7 RNA polymerase, the second promoter, pT7, is activated and is responsible for expressing Selera-2 (Figura 5B).

**FIGURE 6:**
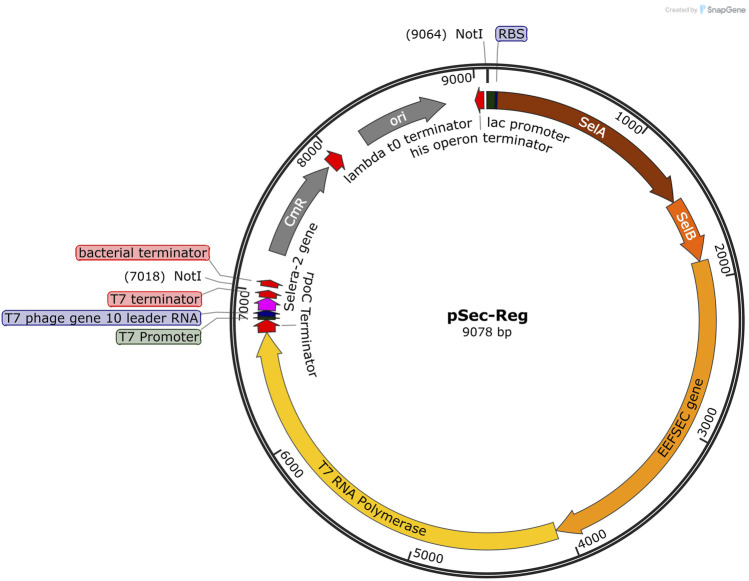
Circular map of plasmid pSec-Reg (9.078 bp). The synthetic plasmid pSec-Reg features a lac promoter for the expression of SelA, SelB and tRNA sec, and a T7 promoter for high-level expression of Selera-2 in *Escherichia coli*. The plasmid map illustrates the presence of the lac promoter (dark green), RBS (dark blue), SelA gene (brown), SelB gene (dark orange), EEFSEC gene (light orange), the T7 RNA polymerase gene (yellow), rpoC Terminator (red), T7 promoter (dark green), RBS - T7 phage gene 10 leader RNA - (dark blue), synthetic Selera-2 sequence (magenta), a T7 terminator (red), origin of replication (ori) (gray), Chloramphenicol resistance protein (Cmr) (gray), and bacterial terminator (red). Additionally, the Not I restriction sites are indicated on the map. The map was visualized using SnapGene software.

The two inserts were concatenated in Benchling, forming a 7,051 bp cassette. This cassette was then virtually digested with the Not I restriction enzyme to create cohesive ends. These ends facilitated the cloning of the cassette into the pS1C3 plasmid, which had also been digested with Not I. The pSec-Reg system ensures that selenoprotein expression in *E. coli* occurs only in the presence of SelA, SelB, and tRNA sec (Figure 6). The plasmid has not yet been *in vitro* constructed. Future work will include validation of the proposed plasmid by verifying expression of the recombinant protein in *E. coli*, Sec incorporation, and its antioxidant properties.

## 4 Discussion

Non-melanoma skin cancer is the most common type of tumor developed by Xeroderma Pigmentosum patients (Lucero and Horowitz, 2023), and the lack of cancerous biomarkers imposes great challenges to therapeutic development (Purkayastha et al., 2023). Most current treatment alternatives include invasive surgeries or topical chemotherapeutics with chronic inflammatory side effects (Hossain et al., 2021). The development of anti-tumoral and antioxidant synergistic biotherapeutics, such as selenopeptides, can be a prominent strategy for addressing XP malignancies.

However, before bench lab *in vitro*/*in vivo* research, it is essential to conduct *in silico* design approaches for ensuring optimal efficacy and viability of recombinant production (Santos et al., 2021), especially for developing selenopeptides (Liu et al., 2018). Selenocysteine (Sec) incorporation requires a system of several proteins alongside the usual translational molecular machinery (Labunskyy et al., 2014), which are fundamental for leveraging recombinant expression yields and also a multistep development pipeline.

The selection of protein sequence templates is the first step for proposing a novel sequence, and since each of the synthetic molecules was intended to be individually customized for antioxidant activity optimization, the array size of five templates was set for feasibility. Peptide templates YR-10, OS-LL11, and LW2 have important structural properties correlated to their ability to induce protection from UV-induced damage (Mirzaei et al., 2019; Xie et al., 2021; Wu et al., 2021); thus, they are a great example of antioxidant activity-based molecules. On the other hand, peptides like Rb4 and LTX-315 play an important role in metabolism regulation, either by overstimulating immune cell response or controlling proliferation (Zhou et al., 2016).

The establishment of rational design is crucial for optimizing functional properties of template proteins, increasing the success potential of new sequences. Incorporation of selenocysteine residues in mid-final positions on the sequence has a greater likelihood of recognition and correct insertion (Shetty et al., 2018), which was a crucial guideline for the sets of Selera sequences designed here. Addition of positively charged residues, like Lysine (K) and Histidine (H), to the sequence was also prioritized as a strategy to improve the antioxidant potential of the molecule, as demonstrated by Tanzadehpanah et al. (2016).

Tertiary structural prediction also contributes to evaluating sequence edition outputs, whether it can be detrimental or optimal for the proposed function (Sahoo and Prasad Burra, 2024). Selera-2 and Selera-3 presented the greatest conserved conformational alpha-helix domain, suggesting that residue modifications were not detrimental to its structures, while the other sequences developed had partial conformational loss, mainly due to charge imbalance disturbance (Zhou and Pang, 2018). Other peptides, such as LTX-315 (Oncopore), currently in clinical trial (NCT06651151) and Dermaseptin-B2, in advanced pre-clinical investigations, also present total positive net charge and amphipathic helical coil structure as important functional features (Camilio et al., 2014; Santos et al., 2017).

Molecular docking is another important step for validating the potential interaction of the designed molecules with the therapeutic targets (Velayutham et al., 2022). Even though there are different peptide-protein docking platforms currently available, peptides have a greater challenge as ligands than small chemical compounds, due to their size, conformational flexibility, and lateral group complexity (Scharbert and Strodel, 2025). Cluspro utilizes the Fast Fourier Transform (FFT) rigid docking program based on ligand rotation and clustering of lowest energy scores to provide high-accuracy models (Kozakov et al., 2017). A flexible docking protocol, CABS-dock (Kurcinski et al., 2015), was also conducted to counteract the rigid docking analysis, since the number of hits generated and scoring function tend to be higher than rigid programs (Desta et al., 2020).

Through docking analysis, Selera-2 demonstrated great potential affinity with both cytosolic targets, B-RAF and TrxR1, suggesting that it might be able to contribute to tumor elimination by inhibiting these targets and their pathways, in addition to intrinsic antioxidant properties (Li et al., 2018; Wang et al., 2024). The stability of these structural docked complexes were also assessed by molecular dynamics simulation, and corroborated the energy scores findings from docking platforms, suggesting that those two models had the most stable binding sites, with overall RMSD <0.3 Å difference (Xin et al., 2024).

Since it had already been demonstrated that peptides, especially with selenium-rich composition, like Selera-2, can act as a synergic coadjuvant for the effective but highly aggressive topical chemotherapeutic imiquimod (Guo et al., 2022), our novel selenopeptide is expected to contribute to its antitumor action and ameliorate the inflammation promoted in a similar mechanism. More specifically, at a cellular level, selenopeptides can act as reactive oxygen species scavengers (Liu et al., 2019) and inhibit or reduce the expression of inflammatory cytokines such as NF-κB, TNF-α, IL-1, IL-6, and IL-8 (Majumder et al., 2016). Nonetheless, unlike synthetic Selera-2, these selenopeptide fractions, as reported by Guo et al. (2020), use no genetic manipulation, and their enzymatic hydrolysis results in a complex mixture, complicating standardization and mechanistic studies.

Access to novel biopharmaceuticals is still challenged by large-scale production complexity and low-yield recombinant expression systems (Jiang et al., 2024). pSec-Reg was proposed in this study to express SelA, SelB, and tRNA^Sec before Selera-2. Mukai et al. (2018) reported a similar expression system that allowed for fine control over expression levels through regulatory signals using variable promoters and arabinose regulation. However, this approach increased the production costs of selenocysteine, in contrast to pSec-Reg, which aims to reduce costs by utilizing classical promoters and a common synthetic biology vector. Miller et al. (2015) developed a synthetic tRNA capable of mediating Sec incorporation in *E. coli* through the canonical EF-Tu pathway. Also, Liu et al. (2018) engineered plasmids encoding orthogonal tRNA/synthetase pairs for the site-specific incorporation of protected or photocaged selenocysteine analogues in *E. coli*. The pSel and pSelExpress1 plasmids (Stanford et al., 2018) have enabled dual expression of the SECIS element and the selenoprotein gene within a single construct, simplifying the biosynthetic pathway.

Despite these advances, existing plasmids often suffer from limitations in host compatibility, expression yield, and folding efficiency, particularly when expressing short synthetic selenopeptides rather than full-length selenoproteins. In recombinant systems, as pSec-Reg was designed, pre-expression of machinery components like SelA, SelB, and tRNA^Sec is required before inducing the protein of interest. This ensures the timely availability of Sec-tRNA^Sec (Cain and Krahn, 2024). pSec-Reg fulfills this need by inducing the expression of Selera-2 or another selenoprotein only after the induction of necessary components (SelA, SelB, and tRNA^Sec) together with T7 RNA polymerase, which activates the activity of the T7 promoter. The novel plasmid construct proposed here integrates optimized regulatory sequences for selenocysteine incorporation, modular cloning sites adaptable to diverse peptide backbones, and elements that improve redox-active peptide folding and yield in *E. coli*.

Expression of native selenoproteins via SECIS/Sel machinery; genetic code expansion (amber suppression) with protected precursors (photocaged Sec) (Peeler and Weerapana, 2022); and use of small molecule antioxidants–NAC (Janeczek et al., 2019), melatonin (Tsujimoto et al., 2025) and photoprotective compounds–T4N5 (Zahid and Brownell, 2008), photolyase (Asahina et al., 1999), afamelanotide (Wu et al., 2024) are methodologies employed to prevent and treat XP. Our approach is an improvement since it is the development of a new pipeline focused on short selenopeptides that offers modularity (easy synthesis/optimization), potential for higher redox activity per mass due to the unique properties of Se, and the possibility of designing target selectivity through structural modeling and docking. However, this advantage can only be confirmed with *in vitro/in vivo* validation compared to current standards.

This study has some limitations. First, the predictions are *in silico* and rely on models and parameterizations that simplify the cellular environment. Second, Sec incorporation models are not a substitute for expression assays and quantification of Sec incorporation, which is why host proteostasis needs to be assessed empirically. Finally, the link between *in vitro* redox activity and *in vivo* therapeutic efficacy (including bioavailability, proteolytic stability, immunogenicity, and dermal uptake) remains to be confirmed. Despite that, implementing *in silico* approaches to validate and develop new compounds, like the one proposed in this work, can optimize time and costs of empirical research, and leverage the success rate of pre-clinical and clinical outcomes (Mehta et al., 2025).

## 5 Conclusion

A new approach for selenoprotein bioengineering towards therapeutic applications was demonstrated through the *in silico* design and testing of the novel selenopeptide Selera-2, which showed promising results for further *in vitro* and pre-clinical investigations of its antitumoral activity and as a potential new treatment strategy for XP and non-melanoma skin cancer tumors. This research design received the Gold Medal and the first runner-up position (second overall) at the iGEM Design League 2023 Competition.

## Data Availability

The datasets presented in this study can be found in online repositories. The names of the repository/repositories and accession number(s) can be found in the article/Supplementary Material.
