# Supplementary material for: Design of synthetic selenopeptides with antioxidant activity for the treatment of XP and non-melanoma skin cancer
Source: Front Syst Biol. 2026 Apr 9;6:1686085. doi: 10.3389/fsysb.2026.1686085 (PMC13102595; doi:10.3389/fsysb.2026.1686085)
Supplement: Supplementary file 1 [file DataSheet2.pdf]

pSec-Reg (9078 bp)

ttgtgagcggataacaattgacattgtgagcggataacaagatactgagcacaaaagaggagaaaatgaccaccgaaacccgcagcctgtatagccagctgccggcg
aacactcgcctattgttaactgtaacactcgcctattgttctatgactcgtgttttctcctcttttactggtggctttgggcgtcggacatatcggtcgacggccgc

lac promoter | RBS | SelA

10 20 30 40 50 60 70 80 90 100

attgatcgcctgctgcgcgatagcagctttctgagcctgcgcgatacctatggccatacccgcgtggtggaactgctgcgccagatgctggatgaagcgcgcgaagt
taactagcggacgacgcgctatcgtcgaaagactcggacgcgctatggataccggtatgggcgcaccaccttgacgacgcggtctacgacctacttcgcgcgcttca

SelA

110 120 130 140 150 160 170 180 190 200 210

gattcgcggcagccagaccctgccggcgtggtgcgaaaactgggcgcaggaagtggatgcgcgcctgaccaaagaagcgcagagcgcgctgcgcccggtgattaacc
ctaagcgccgtcggtctgggacggccgcaccacgcttttgacccgcgtccttcacctacgcgcggactggtttcttcgcgtctcgcgcgacgcgggccactaattgg

SelA

220 230 240 250 260 270 280 290 300 310 320

tgaccggcaccgtgctgcataccaacctgggccgcgcgctgcaggcggaagcggcggtggaagcggtggcgcaggcgatgcgcagcccggtgaccctggaatatgat
actggccgtggcacgacgtatggttggacccggcgcgcgacgtccgccttcgccgccaccttcgccaccgcgtccgctacgcgtcgggccactgggaccttatacta

SelA

330 340 350 360 370 380 390 400 410 420

ctggatgatgcgggccgcggccatcgcgatcgcgcgctggcgcagctgctgtgccgcattaccggcgcggaagatgcgtgcattgtgaacaacaacgcggcggcggt
gacctactacgcccggcgccggtagcgctagcgcgcgaccgcgtcgacgacacggcgtaatggccgcgccttctacgcacgtaacacttgttgttgcgccgccgcca

SelA

430 440 450 460 470 480 490 500 510 520 530

gctgctgatgctggcggcgaccgcgagcggcaaagaagtggtggtgagccgcggcgaactggtggaaattggcggcgcgtttcgcattccggatgtgatgcgccagg
cgacgactacgaccgccgctggcgctcgccgtttcttcaccaccactcggcgccgcttgaccacctttaaccgccgcgcaaagcgtaaggcctacactacgcggtcc

SelA

540 550 560 570 580 590 600 610 620 630 640

cgggctgcaccctgcatgaagtgggcaccaccaaccgcacccatgcgaacgattatcgccaggcggtgaacgaaaacaccgcgctgctgatgaaagtgcataccagc
gcccgacgtgggacgtacttcacccgtggtggttggcgtgggtacgcttgctaatagcggtccgccacttgcttttgtggcgcgacgactactttcacgtatggtcg

SelA

650 660 670 680 690 700 710 720 730 740

aactatagcattcagggctttaccaaagcgattgatgaagcggaactggtggcgctgggcaaagaactggatgtgccggtggtgaccgatctgggcagcggcagcct
ttgatatcgtaagtcccgaaatggtttcgctaactacttcgccttgaccaccgcgacccgtttcttgacctacacggccaccactggctagacccgtcgccgtcgga

SelA

750 760 770 780 790 800 810 820 830 840 850

ggtggatctgagccagtatggcctgccgaaagaaccgatgccgcaggaactgattgcggcgggcgtgagcctggtgagctttagcggcgataaactgctgggcggcc
ccacctagactcggtcataccggacggctttcttggctacggcgtccttgactaacgccgcccgcactcggaccactcgaaatcgccgctatttgacgacccgccgg
SelA
860 870 880 890 900 910 920 930 940 950 960

cgcaggcgggcattattgtgggcaaaaaagaaatgattgcgcgcctgcagagccatccgctgaaacgcgcgctgcgcgcggataaaatgaccctggcggcgctggaa
gcgtccgcccgtaataacacccgttttttctttactaacgcgcggacgtctcggtaggcgactttgcgcgcgacgcgcgcctattttactgggaccgccgcgacctt
SelA
970 980 990 1,000 1,010 1,020 1,030 1,040 1,050 1,060 1,070

gcgaccctgcgcctgtatctgcatccggaagcgctgagcgaaaaactgccgaccctgcgcctgctgacccgcagcgcggaagtgattcagattcaggcgcagcgcct
cgctgggacgcggacatagacgtaggccttcgcgactcgcttttgacggctgggacgcggacgactgggcgtcgcgccttcactaagtctaagtccgcgtcgcgga
SelA
1,080 1,090 1,100 1,110 1,120 1,130 1,140 1,150 1,160 1,170

gcaggcgccgctggcggcgcattatggcgcggaatttgcggtgcaggtgatgccgtgcctgagccagattggcagcggcagcctgccggtggatcgcctgccgagcg
cgtccgcggcgaccgccgcgtaataccgcgccttaaacgccacgtccactacggcacggactcggtctaaccgtcgccgtcggacggccacctagcggacggctcgc
SelA
1,180 1,190 1,200 1,210 1,220 1,230 1,240 1,250 1,260 1,270 1,280

cggcgctgacctttaccccgcatgatggccgcggcagccatctggaaagcctggcggcgcgctggcgcgaactgccggtgccggtgattggccgcatttatgatggc
gccgcgactggaaatggggcgtactaccggcgccgtcggtagacctttcggaccgccgcgcgaccgcgcttgacggccacggccactaaccggcgtaaatactaccg
SelA
1,290 1,300 1,310 1,320 1,330 1,340 1,350 1,360 1,370 1,380 1,390

cgcctgtggctggatctgcgctgcctggaagatgaacagcgctttctggaaatgctgctgaaaMNNATAGHVDHGKTTANTGVNADRKKRGMTNDGYAYWDGRVGND
gcggacaccgacctagacgcgacggaccttctacttgtcgcgaaagacctttacgacgactttKNNTATCDBHDCMAATNACBNTHYMMYCKANHCRTRWHCYBCNH
SelA selB
1,400 1,410 1,420 1,430 1,440 1,450 1,460 1,470 1,480 1,490

VGHKSNMAGVGGNDHAVVACDDGVMATRHANTGNMTVATKADRVDARVDVRVKVRYGAAKNTAATGRGMDARHRHASHSRANDRATVKGAGVVTGTASGVKVGDSWT
BCDMSNKTCBCCNHDTBBTGHHCBKTAYDTNACNKABTAMTHYBHTYBHBYBMBYRCTTMNATTACYCKHTYDYDTSDSYTNHYTABMCTCBBACATSCBMBCHSWA
selB
1,500 1,510 1,520 1,530 1,540 1,550 1,560 1,570 1,580 1,590 1,600

GVNKMRVRAHANTTANAGRNANNAGDAKNNRGDWADVTRVNVTHTTWHNHHAASHVTGRVSDNAVDTWADNDRVRDNSARNTAGARVVMNRRGKRKYWASARASDAD
CBNMKYBYTDTNAATNTCYNTNNTCHTMNNYCHWTHBAYBNBADAAWDNDDTTSDBACYBSHNTBHAWTHNHYBYHNSTYNATCTYBBKNYYCMYMRWTSTYTSHTH
selB
1,610 1,620 1,630 1,640 1,650 1,660 1,670 1,680 1,690 1,700 1,710

ASVHRGAVNADAWARNGGMRGYNAGYSNAVAARWRKNDTATYHHRDGGRRRRMAMDAVNKMRSGDNHSHHGWHDHKAGSANWKAGDWWVRDAKTGTDAMRTRAAGNN
TSBDYCTBNTHTWTYNCCKYCRNTCRSNTBTTYWYMNHATARDDYHCCYYYYKTKHTBNMKYSCHNDSDDCWDHDMTCSTNWMTCHWWBYHTMACAHTKYAYTTCNN
selB
1,720 1,730 1,740 1,750 1,760 1,770 1,780 1,790 1,800 1,810

TANVKDRYYRNDRNVANMNRDDCGSTCAADRDRGVGRKANNYDRNGTRRRGNDHRDAKgcgggtgtccgaggcggcggcggcggcatggcagggcggcgggtgaacg
ATNBMHYRRYNHYNBTNKNYHHGCSAGTTHYHYCBCYMTNNRHYNCAYYYCNHDYHTMcgcccacaggctccgccgccgccgccgtaccgtcccgccgcccacttgc
selB EEFSEC gene
1,820 1,830 1,840 1,850 1,860 1,870 1,880 1,890 1,900 1,910 1,920

tgaacgtgggcgtgctgggccacatcgacagcggcaagacggcgctggcgcgggcgctaagcaccacagcctccaccgccgcctttgacaagcagccgcagagccgc
acttgcacccgcacgacccggtgtagctgtcgccgttctgccgcgaccgcgcccgcgattcgtggtgtcggaggtggcggcggaaactgttcgtcggcgtctcggcg
EEFSEC gene
1,930 1,940 1,950 1,960 1,970 1,980 1,990 2,000 2,010 2,020 2,030

gagcgcggcatcacgctcgatctgggcttctcgtgcttctcggtgccgctgcccgcgcgcctgcggtcgtctttgcccgagttccaggcagcgcccgaggccgagcc
ctcgcgccgtagtgcgagctagacccgaagagcacgaagagccacggcgacgggcgcgcggacgccagcagaaacgggctcaaggtccgtcgcgggctccggctcgg
EEFSEC gene
2,040 2,050 2,060 2,070 2,080 2,090 2,100 2,110 2,120 2,130 2,140

cgagcccggcgagccactgcttcaggtcacgctggtcgactgccccgggcacgcctccctcatccggaccatcatcggcggggcccagatcattgatctgatgatgc
gctcgggccgctcggtgacgaagtccagtgcgaccagctgacggggcccgtgcggagggagtaggcctggtagtagccgccccgggtctagtaactagactactacg
EEFSEC gene
2,150 2,160 2,170 2,180 2,190 2,200 2,210 2,220 2,230 2,240

tggtcatcgatgtgaccaaggggatgcagacccagtcagcggaatgccttgtgatcggccagattgcctgccagaagctggtcgtggtgctgaacaaaatagacctc
accagtagctacactggttcccctacgtctgggtcagtcgccttacggaacactagccggtctaacggacggtcttcgaccagcaccacgacttgttttatctggag
EEFSEC gene
2,250 2,260 2,270 2,280 2,290 2,300 2,310 2,320 2,330 2,340 2,350

ttacctgaaggaaagagacaggcagcaattgataaaatgaccaagaaaatgcagaagaccctagagaacaccaagttccgaggtgcaccgattatacccgtggcggc
aatggacttcctttctctgtccgtcgttaactattttactggttcttttacgtcttctgggatctcttgtggttcaaggctccacgtggctaatatgggcaccgccg
EEFSEC gene
2,360 2,370 2,380 2,390 2,400 2,410 2,420 2,430 2,440 2,450 2,460

caagccgggggggaccagaggcccccgaaactgaagctccacagggcattccagagctcattgagctcctgacgtcccagatttccatcccaacgagagatccctcgg
gttcggccccccctggtctccgggggctttgacttcgaggtgtcccgtaaggtctcgagtaactcgaggactgcagggtctaaaggtagggttgctctctagggagcc
EEFSEC gene
2,470 2,480 2,490 2,500 2,510 2,520 2,530 2,540 2,550 2,560

gaccgttcctcatgtctgtggaccactgtttctccatcaaaggccaaggcactgtgatgacagggaccatcctttcaggctccatcagcctcggtgacagtgtggag
ctggcaaggagtacagacacctggtgacaaagaggtagtttccggttccgtgacactactgtccctggtaggaaagtccgaggtagtcggagccactgtcacacctc
EEFSEC gene
2,570 2,580 2,590 2,600 2,610 2,620 2,630 2,640 2,650 2,660 2,670

atccctgccctcaaggtggtgaagaaggtgaagtccatgcagatgttccacatgcccatcacttcagccatgcaaggagaccggctgggcatctgcgtcacccagtt
tagggacgggagttccaccacttcttccacttcaggtacgtctacaaggtgtacgggtagtgaagtcggtacgttcctctggccgacccgtagacgcagtgggtcaa
EEFSEC gene
2,680 2,690 2,700 2,710 2,720 2,730 2,740 2,750 2,760 2,770 2,780

tgaccctaagctgctggagcgcgggttggtgtgtgcccccgagtccctgcacactgtccatgcggccctcatctctgtggaaaagataccgtatttccgggggcccc
actgggattcgacgacctcgcgcccaaccacacacgggggctcagggacgtgtgacaggtacgccgggagtagagacaccttttctatggcataaaggcccccgggg
EEFSEC gene
2,790 2,800 2,810 2,820 2,830 2,840 2,850 2,860 2,870 2,880

tgcaaaccaaggccaagttccacattacagtgggccatgaaacagtcatgggccggttgatgttcttcagtcctgctccagataactttgaccaggagcctatactg
acgtttggttccggttcaaggtgtaatgtcacccggtactttgtcagtacccggccaactacaagaagtcaggacgaggtctattgaaactggtcctcggatatgac
EEFSEC gene
2,890 2,900 2,910 2,920 2,930 2,940 2,950 2,960 2,970 2,980 2,990

gactctttcaacttctctcaagaataccttttccaggagcagtacctgtccaaggatttgacaccagcagtgacagacaatgatgaggccgacaagaaggccggcca
ctgagaaagttgaagagagttcttatggaaaaggtcctcgtcatggacaggttcctaaactgtggtcgtcactgtctgttactactccggctgttcttccggccggt
EEFSEC gene
3,000 3,010 3,020 3,030 3,040 3,050 3,060 3,070 3,080 3,090 3,100

ggccacagagggccattgtcctcggcagcagtgggccctggtggagtttgagaagcccgtcacctgccctcggctgtgcctggtgattggctccaggctagatgcgg
ccggtgtctcccggtaacaggagccgtcgtcacccgggaccacctcaaactcttcgggcagtggacgggagccgacacggaccactaaccgaggtccgatctacgcc
EEFSEC gene
3,110 3,120 3,130 3,140 3,150 3,160 3,170 3,180 3,190 3,200 3,210

acattcacaccaacacgtgccggctagccttccatggcatcctgctccacgggctagaggacaggaactacgccgacagcttcctgcccaggctgaaggtgtacaag
tgtaagtgtggttgtgcacggccgatcggaaggtaccgtaggacgaggtgcccgatctcctgtccttgatgcggctgtcgaaggacgggtccgacttccacatgttc
EEFSEC gene
3,220 3,230 3,240 3,250 3,260 3,270 3,280 3,290 3,300 3,310

ctgaagcacaagcatggccttgtggagcgggcgatggatgactacagtgtgatcggccgctccctgttcaaaaaggaaaccaacatccagctcttcgtggggctcaa
gacttcgtgttcgtaccggaacacctcgcccgctacctactgatgtcacactagccggcgagggacaagtttttcctttggttgtaggtcgagaagcaccccgagtt
EEFSEC gene
3,320 3,330 3,340 3,350 3,360 3,370 3,380 3,390 3,400 3,410 3,420

ggtgcacttgtccactggggaactgggcatcatcgacagtgccttcggccagagcggcaagttcaagatccacatcccaggtggcctcagccccgagtccaagaaga
ccacgtgaacaggtgaccccttgacccgtagtagctgtcacggaagccggtctcgccgttcaagttctaggtgtagggtccaccggagtcggggctcaggttcttct
EEFSEC gene
3,430 3,440 3,450 3,460 3,470 3,480 3,490 3,500 3,510 3,520 3,530

tcctgacacccgccctcaagaagcgggcccgggctggccgtggggaggccaccaggcaggaggagagcgccgagcggagcgagccctcacagcatgtggtgctcagc
aggactgtgggcgggagttcttcgcccgggcccgaccggcacccctccggtggtccgtcctcctctcgcggctcgcctcgctcgggagtgtcgtacaccacgagtcg
EEFSEC gene
3,540 3,550 3,560 3,570 3,580 3,590 3,600 3,610 3,620 3,630

ctgactttcaagcgttatgtcttcgacacccacaagcgcatggttcagtctccctgagtgtccggtgacctcccccagggcctccttgcccagcccagtccaggctg
gactgaaagttcgcaatacagaagctgtgggtgttcgcgtaccaagtcagagggactcacaggccactggagggggtcccggaggaacgggtcgggtcaggtccgac
EEFSEC gene
3,640 3,650 3,660 3,670 3,680 3,690 3,700 3,710 3,720 3,730 3,740

ctgtgccaaatcccaaccagccacgcctcagcctctcccagtctctccctgcagtcctgcagcagcagcccccacccccaagcttggtgctgagccctggtgaggag
gacacggtttagggttggtcggtgcggagtcggagagggtcagagagggacgtcaggacgtcgtcgtcgggggtgggggttcgaaccacgactcgggaccactcctc
EEFSEC gene
3,750 3,760 3,770 3,780 3,790 3,800 3,810 3,820 3,830 3,840 3,850

ctgagggggatgggttgctggggccaggagggtctctcctccagcccctgcacactcccacccaggacagcccccagcccaactaggaaagggccatgggcagaggg
gactccccctacccaacgaccccggtcctcccagagaggaggtcggggacgtgtgagggtgggtcctgtcgggggtcgggttgatcctttcccggtacccgtctccc
EEFSEC gene
3,860 3,870 3,880 3,890 3,900 3,910 3,920 3,930 3,940 3,950

ctggtagccagtatcttccactgccccatctgttggccacctgcaggccagtctcaaccctcccccaggtgggcaggcacttgatggctacaaataaatgtcccgtg
gaccatcggtcatagaaggtgacggggtagacaaccggtggacgtccggtcagagttgggagggggtccacccgtccgtgaactaccgatgtttatttacagggcac
EEFSEC gene
3,960 3,970 3,980 3,990 4,000 4,010 4,020 4,030 4,040 4,050 4,060

gccccagcccactctaatgaacacgattaacatcgctaagaacgacttctctgacatcgaactggctgctatcccgttcaacactctggctgaccattacggtgagc
cggggtcgggtgagattacttgtgctaattgtagcgattcttgctgaagagactgtagcttgaccgacgatagggcaagttgtgagaccgactggtaatgccactcg
EEFSEC gene T7 RNA Polymerase
4,070 4,080 4,090 4,100 4,110 4,120 4,130 4,140 4,150 4,160 4,170

gtttagctcgcgaacagttggcccttgagcatgagtcttacgagatgggtgaagcacgcttccgcaagatgtttgagcgtcaacttaaagctggtgaggttgcggat
caaatcgagcgcttgtcaaccgggaactcgtactcagaatgctctacccacttcgtgcgaaggcgttctacaaactcgcagttgaatttcgaccactccaacgccta
T7 RNA Polymerase
4,180 4,190 4,200 4,210 4,220 4,230 4,240 4,250 4,260 4,270 4,280

aacgctgccgccaagcctctcatcactaccctactccctaagatgattgcacgcatcaacgactggtttgaggaagtgaaagctaagcgcggcaagcgcccgacagc
ttgcgacggcggttcggagagtagtgatgggatgagggattctactaacgtgcgtagttgctgaccaaactccttcactttcgattcgcgccgttcgcgggctgtcg

T7 RNA Polymerase

4,290 4,300 4,310 4,320 4,330 4,340 4,350 4,360 4,370 4,380

cttccagttcctgcaagaaatcaagccggaagccgtagcgtacatcaccattaagaccactctggcttgcctaaccagtgctgacaatacaaccgttcaggctgtag
gaaggtcaaggacgttctttagttcggccttcggcatcgcatgtagtggtaattctggtgagaccgaacggattggtcacgactgttatgttggcaagtccgacatc

T7 RNA Polymerase

4,390 4,400 4,410 4,420 4,430 4,440 4,450 4,460 4,470 4,480 4,490

caagcgcaatcggtcgggccattgaggacgaggctcgcttcggtcgtatccgtgaccttgaagctaagcacttcaagaaaaacgttgaggaacaactcaacaagcgc
gttcgcgttagccagcccggtaactcctgctccgagcgaagccagcataggcactggaacttcgattcgtgaagttctttttgcaactccttgttgagttgttcgcg

T7 RNA Polymerase

4,500 4,510 4,520 4,530 4,540 4,550 4,560 4,570 4,580 4,590 4,600

gtagggcacgtctacaagaaagcatttatgcaagttgtcgaggctgacatgctctctaagggtctactcggtggcgaggcgtggtcttcgtggcataaggaagactc
catcccgtgcagatgttctttcgtaaatacgttcaacagctccgactgtacgagagattcccagatgagccaccgctccgcaccagaagcaccgtattccttctgag

T7 RNA Polymerase

4,610 4,620 4,630 4,640 4,650 4,660 4,670 4,680 4,690 4,700

tattcatgtaggagtacgctgcatcgagatgctcattgagtcaaccggaatggttagcttacaccgccaaaatgctggcgtagtaggtcaagactctgagactatcg
ataagtacatcctcatgcgacgtagctctacgagtaactcagttggccttaccaatcgaatgtggcggttttacgaccgcatcatccagttctgagactctgatagc

T7 RNA Polymerase

4,710 4,720 4,730 4,740 4,750 4,760 4,770 4,780 4,790 4,800 4,810

aactcgcacctgaatacgctgaggctatcgcaacccgtgcaggtgcgctggctggcatctctccgatgttccaaccttgcgtagttcctcctaagccgtggactggc
ttgagcgtggacttatgcgactccgatagcgttgggcacgtccacgcgaccgaccgtagagaggctacaaggttggaacgcatcaaggaggattcggcacctgaccg

T7 RNA Polymerase

4,820 4,830 4,840 4,850 4,860 4,870 4,880 4,890 4,900 4,910 4,920

attactggtggtggctattgggctaacggtcgtcgtcctctggcgctggtgcgtactcacagtaagaaagcactgatgcgctacgaagacgtttacatgcctgaggt
taatgaccaccaccgataacccgattgccagcagcaggagaccgcgaccacgcatgagtgtcattctttcgtgactacgcgatgcttctgcaaatgtacggactcca

T7 RNA Polymerase

4,930 4,940 4,950 4,960 4,970 4,980 4,990 5,000 5,010 5,020

gtacaaagcgattaacattgcgcaaaacaccgcatggaaaatcaacaagaaagtcctagcggtcgccaacgtaatcaccaagtggaagcattgtccggtcgaggaca
catgtttcgctaattgtaacgcgttttgtggcgtaccttttagttgttctttcaggatcgccagcggttgcattagtggttcaccttcgtaacaggccagctcctgt

T7 RNA Polymerase

5,030 5,040 5,050 5,060 5,070 5,080 5,090 5,100 5,110 5,120 5,130

tccctgcgattgagcgtgaagaactcccgatgaaaccggaagacatcgacatgaatcctgaggctctcaccgcgtggaaacgtgctgccgctgctgtgtaccgcaag
agggacgctaactcgcacttcttgagggctactttggccttctgtagctgtacttaggactccgagagtggcgcacctttgcacgacggcgacgacacatggcgttc
T7 RNA Polymerase
5,140 5,150 5,160 5,170 5,180 5,190 5,200 5,210 5,220 5,230 5,240

gacagggctcgcaagtctcgccgtatcagccttgagttcatgcttgagcaagccaataagtttgctaaccataaggccatctggttcccttacaacatggactggcg
ctgtcccgagcgttcagagcggcatagtcggaactcaagtacgaactcgttcggttattcaaacgattggtattccggtagaccaagggaatgttgtacctgaccgc
T7 RNA Polymerase
5,250 5,260 5,270 5,280 5,290 5,300 5,310 5,320 5,330 5,340 5,350

cggtcgtgtttacgccgtgtcaatgttcaacccgcaaggtaacgatatgaccaaaggactgcttacgctggcgaaaggtaaaccaatcggtaaggaaggttactact
gccagcacaaatgcggcacagttacaagttgggcgttccattgctatactggtttcctgacgaatgcgaccgctttccatttggttagccattccttccaatgatga
T7 RNA Polymerase
5,360 5,370 5,380 5,390 5,400 5,410 5,420 5,430 5,440 5,450

ggctgaaaatccacggtgcaaactgtgcgggtgtcgataaggttccgttccctgagcgcatcaagttcattgaggaaaaccacgagaacatcatggcttgcgctaag
ccgacttttaggtgccacgtttgacacgcccacagctattccaaggcaagggactcgcgtagttcaagtaactccttttggtgctcttgtagtaccgaacgcgattc
T7 RNA Polymerase
5,460 5,470 5,480 5,490 5,500 5,510 5,520 5,530 5,540 5,550 5,560

tctccactggagaacacttggtgggctgagcaagattctccgttctgcttccttgcgttctgctttgagtacgctggggtacagcaccacggcctgagctataactg
agaggtgacctcttgtgaaccacccgactcgttctaagaggcaagacgaaggaacgcaagacgaaactcatgcgaccccatgtcgtggtgccggactcgatattgac
T7 RNA Polymerase
5,570 5,580 5,590 5,600 5,610 5,620 5,630 5,640 5,650 5,660 5,670

ctcccttccgctggcgtttgacgggtcttgctctggcatccagcacttctccgcgatgctccgagatgaggtaggtggtcgcgcggttaacttgcttcctagtgaga
gagggaaggcgaccgcaaactgcccagaacgagaccgtaggtcgtgaagaggcgctacgaggctctactccatccaccagcgcgccaattgaacgaaggatcactct
T7 RNA Polymerase
5,680 5,690 5,700 5,710 5,720 5,730 5,740 5,750 5,760 5,770

ccgttcaggacatctacgggattgttgctaagaaagtcaacgagattctacaagcagacgcaatcaatgggaccgataacgaagtagttaccgtgaccgatgagaac
ggcaagtcctgtagatgccctaacaacgattctttcagttgctctaagatgttcgtctgcgttagttaccctggctattgcttcatcaatggcactggctactcttg
T7 RNA Polymerase
5,780 5,790 5,800 5,810 5,820 5,830 5,840 5,850 5,860 5,870 5,880

actggtgaaatctctgagaaagtcaagctgggcactaaggcactggctggtcaatggctggctcacggtgttactcgcagtgtgactaagcgttcagtcatgacgct
tgaccactttagagactctttcagttcgacccgtgattccgtgaccgaccagttaccgaccgagtgccacaatgagcgtcacactgattcgcaagtcagtactgcga
T7 RNA Polymerase
5,890 5,900 5,910 5,920 5,930 5,940 5,950 5,960 5,970 5,980 5,990

```
ggcttacgggtccaaagagttcggcttccgtcaacaagtgctggaagataccattcagccagctattgattccggcaagggtccgatgttcactcagccgaatcagg
ccgaatgcccaggtttctcaagccgaaggcagttgttcacgaccttctatggtaagtcggtcgataactaaggccgttcccaggctacaagtgagtcggcttagtcc
                                   T7 RNA Polymerase
    6,000     6,010     6,020     6,030     6,040     6,050     6,060     6,070     6,080     6,090

ctgctggatacatggctaagctgatttgggaatctgtgagcgtgacggtggtagctgcggttgaagcaatgaactggcttaagtctgctgctaagctgctggctgct
gacgacctatgtaccgattcgactaaacccttagacactcgcactgccaccatcgacgccaacttcgttacttgaccgaattcagacgacgattcgacgaccgacga
                                   T7 RNA Polymerase
6,100  6,110     6,120     6,130     6,140     6,150     6,160     6,170     6,180     6,190     6,200

gaggtcaaagataagaagactggagagattcttcgcaagcgttgcgctgtgcattgggtaactcctgatggtttccctgtgtggcaggaatacaagaagcctattca
ctccagtttctattcttctgacctctctaagaagcgttcgcaacgcgacacgtaacccattgaggactaccaaagggacacaccgtccttatgttcttcggataagt
                                   T7 RNA Polymerase
6,210     6,220     6,230     6,240     6,250     6,260     6,270     6,280     6,290     6,300     6,310

gacgcgcttgaacctgatgttcctcggtcagttccgcttacagcctaccattaacaccaacaaagatagcgagattgatgcacacaaacaggagtctggtatcgctc
ctgcgcgaacttggactacaaggagccagtcaaggcgaatgtcggatggtaattgtggttgtttctatcgctctaactacgtgtgtttgtcctcagaccatagcgag
                                   T7 RNA Polymerase
   6,320     6,330     6,340     6,350     6,360     6,370     6,380     6,390     6,400     6,410   6,420

ctaactttgtacacagccaagacggtagccaccttcgtaagactgtagtgtgggcacacgagaagtacggaatcgaatcttttgcactgattcacgactccttcggt
gattgaaacatgtgtcggttctgccatcggtggaagcattctgacatcacacccgtgtgctcttcatgccttagcttagaaaacgtgactaagtgctgaggaagcca
                                   T7 RNA Polymerase
      6,430     6,440     6,450     6,460     6,470     6,480     6,490     6,500     6,510     6,520

accattccggctgacgctgcgaacctgttcaaagcagtgcgcgaaactatggttgacacatatgagtcttgtgatgtactggctgatttctacgaccagttcgctga
tggtaaggccgactgcgacgcttggacaagtttcgtcacgcgctttgataccaactgtgtatactcagaacactacatgaccgactaaagatgctggtcaagcgact
                                   T7 RNA Polymerase
6,530     6,540     6,550     6,560     6,570     6,580     6,590     6,600     6,610     6,620     6,630

ccagttgcacgagtctcaattggacaaaatgccagcacttccggctaaaggtaacttgaacctccgtgacatcttagagtcggacttcgcgttcgcgtaagtaatcg
ggtcaacgtgctcagagttaacctgttttacggtcgtgaaggccgatttccattgaacttggaggcactgtagaatctcagcctgaagcgcaagcgcattcattagc
                                T7 RNA Polymerase
  6,640     6,650     6,660     6,670     6,680     6,690     6,700     6,710     6,720     6,730   6,740

ttaatccgcaaataacgtaaaaacccgcttcggcgggtttttttatgggggagtttagggaaagagcatttgtcaTAATACGACTCACTATAGTTCGAAAATTAAT
aattaggcgtttattgcatttttgggcgaagccgcccaaaaaaatacccccctcaaatccctttctcgtaaacagtATTATGCTGAGTGATATCAAGCTTTTAATTA
                      rpoC Terminator                             T7 promoter       T7 ...NA
    6,750     6,760     6,770     6,780     6,790     6,800     6,810     6,820     6,830     6,840
```

```
ACGACTCACTATAGGGAGACCACAACGGatggccgattcggaacgcctgcatcatagcgcacacaagaaaggctgggcggcgtgcacgaacttttcacgttgtaccc
TGCTGAGTGATATCCCTCTGGTGTTGCCtaccggctaagccttgcggacgtagtatcgcgtgtgttctttccgacccgccgcacgtgcttgaaaagtgcaacatggg
```

T7 phage ge...leader RNA | Selera-2

6,850 6,860 6,870 6,880 6,890 6,900 6,910 6,920 6,930 6,940 6,950

NotI

```
gcaaactagcataaccccttggggcctctaaacgggtcttgaggggttttttgTACTAGTAGCGGCCGCGAATTCCAGAAATCATCCTTAGCGAAAGCTAAGGATTT
cgtttgatcgtattggggaaccccggagatttgcccagaactccccaaaaaacATGATCATCGCCGGCGCTTAAGGTCTTTAGTAGGAATCGCTTTCGATTCCTAAA
```

T7 terminator | Bio...ix | bacterial terminator

6,960 6,970 6,980 6,990 7,000 7,010 7,020 7,030 7,040 7,050 7,060

```
TTTTTATCTGAAATTCTGCCTCGTGATACGCCTATTTTTATAGGTTAATGTCATGATAATAATGGTTTCTTAGACGTCAGGTGGCACTCGAGTTGATCGGGCACGTA
AAAAATAGACTTTAAGACGGAGCACTATGCGGATAAAAATATCCAATTACAGTACTATTATTACCAAAGAATCTGCAGTCCACCGTGAGCTCAACTAGCCCGTGCAT
```

bact...tor

7,070 7,080 7,090 7,100 7,110 7,120 7,130 7,140 7,150 7,160

```
AGAGGTTCCAACTTTCACCATAATGAAATAAGATCACTACCGGGCGTATTTTTTGAGTTATCGAGATTTTCAGGAGCTAAGGAAGCTAAAATGGAGAAAAAAATCAC
TCTCCAAGGTTGAAAGTGGTATTACTTTATTCTAGTGATGGCCCGCATAAAAAACTCAATAGCTCTAAAAGTCCTCGATTCCTTCGATTTTACCTCTTTTTTTAGTG
```

CmR

7,170 7,180 7,190 7,200 7,210 7,220 7,230 7,240 7,250 7,260 7,270

```
TGGATATACCACCGTTGATATATCCCAATGGCATCGTAAAGAACATTTTGAGGCATTTCAGTCAGTTGCTCAATGTACCTATAACCAGACCGTTCAGCTGGATATTA
ACCTATATGGTGGCAACTATATAGGGTTACCGTAGCATTTCTTGTAAAACTCCGTAAAGTCAGTCAACGAGTTACATGGATATTGGTCTGGCAAGTCGACCTATAAT
```

CmR

7,280 7,290 7,300 7,310 7,320 7,330 7,340 7,350 7,360 7,370 7,380

```
CGGCCTTTTTAAAGACCGTAAAGAAAAATAAGCACAAGTTTTATCCGGCCTTTATTCACATTCTTGCCCGCCTGATGAATGCTCATCCGGAATTTCGTATGGCAATG
GCCGGAAAAATTTCTGGCATTTCTTTTTATTCGTGTTCAAAATAGGCCGGAAATAAGTGTAAGAACGGGCGGACTACTTACGAGTAGGCCTTAAAGCATACCGTTAC
```

CmR

7,390 7,400 7,410 7,420 7,430 7,440 7,450 7,460 7,470 7,480 7,490

```
AAAGACGGTGAGCTGGTGATATGGGATAGTGTTCACCCTTGTTACACCGTTTTCCATGAGCAAACTGAAACGTTTTCATCGCTCTGGAGTGAATACCACGACGATTT
TTTCTGCCACTCGACCACTATACCCTATCACAAGTGGGAACAATGTGGCAAAAGGTACTCGTTTGACTTTGCAAAAGTAGCGAGACCTCACTTATGGTGCTGCTAAA
```

CmR

7,500 7,510 7,520 7,530 7,540 7,550 7,560 7,570 7,580 7,590

```
CCGGCAGTTTCTACACATATATTCGCAAGATGTGGCGTGTTACGGTGAAAACCTGGCCTATTTCCCTAAAGGGTTTATTGAGAATATGTTTTTCGTCTCAGCCAATC
GGCCGTCAAAGATGTGTATATAAGCGTTCTACACCGCACAATGCCACTTTTGGACCGGATAAAGGGATTTCCCAAATAACTCTTATACAAAAAGCAGAGTCGGTTAG
```

CmR

7,600 7,610 7,620 7,630 7,640 7,650 7,660 7,670 7,680 7,690 7,700

CCTGGGTGAGTTTCACCAGTTTTGATTTAAACGTGGCCAATATGGACAACTTCTTCGCCCCCGTTTTCACCATGGGCAAATATTATACGCAAGGCGACAAGGTGCTG
GGACCCACTCAAAGTGGTCAAAACTAAATTTGCACCGGTTATACCTGTTGAAGAAGCGGGGGCAAAAGTGGTACCCGTTTATAATATGCGTTCCGCTGTTCCACGAC

CmR

7,710 7,720 7,730 7,740 7,750 7,760 7,770 7,780 7,790 7,800 7,810

ATGCCGCTGGCGATTCAGGTTCATCATGCCGTTTGTGATGGCTTCCATGTCGGCAGAATGCTTAATGAATTACAACAGTACTGCGATGAGTGGCAGGGCGGGGCGTA
TACGGCGACCGCTAAGTCCAAGTAGTACGGCAAACACTACCGAAGGTACAGCCGTCTTACGAATTACTTAATGTTGTCATGACGCTACTCACCGTCCCGCCCCGCAT

CmR

7,820 7,830 7,840 7,850 7,860 7,870 7,880 7,890 7,900 7,910

ATTTGATATCGAGCTCGCTTGGACTCCTGTTGATAGATCCAGTAATGACCTCAGAACTCCATCTGGATTTGTTCAGAACGCTCGGTTGCCGCCGGGCGTTTTTTATT
TAAACTATAGCTCGAGCGAACCTGAGGACAACTATCTAGGTCATTACTGGAGTCTTGAGGTAGACCTAAACAAGTCTTGCGAGCCAACGGCGGCCCGCAAAAAATAA

lambda t0 terminator

7,920 7,930 7,940 7,950 7,960 7,970 7,980 7,990 8,000 8,010 8,020

GGTGAGAATCCAAGCCTCGAGCTGTCAGACCAAGTTTACTCATATATACTTTAGATTGATTTAAAACTTCATTTTTAATTTAAAAGGATCTAGGTGAAGATCCTTTT
CCACTCTTAGGTTCGGAGCTCGACAGTCTGGTTCAAATGAGTATATATGAAATCTAACTAAATTTTGAAGTAAAAATTAAATTTTCCTAGATCCACTTCTAGGAAAA

8,030 8,040 8,050 8,060 8,070 8,080 8,090 8,100 8,110 8,120 8,130

TGATAATCTCATGACCAAAATCCCTTAACGTGAGTTTTCGTTCCACTGAGCGTCAGACCCCGTAGAAAAGATCAAAGGATCTTCTTGAGATCCTTTTTTTCTGCGCG
ACTATTAGAGTACTGGTTTTAGGGAATTGCACTCAAAAGCAAGGTGACTCGCAGTCTGGGGCATCTTTTCTAGTTTCCTAGAAGAACTCTAGGAAAAAAAGACGCGC

ori

8,140 8,150 8,160 8,170 8,180 8,190 8,200 8,210 8,220 8,230

TAATCTGCTGCTTGCAAACAAAAAAACCACCGCTACCAGCGGTGGTTTGTTTGCCGGATCAAGAGCTACCAACTCTTTTTCCGAAGGTAACTGGCTTCAGCAGAGCG
ATTAGACGACGAACGTTTGTTTTTTTGGTGGCGATGGTCGCCACCAAACAAACGGCCTAGTTCTCGATGGTTGAGAAAAAGGCTTCCATTGACCGAAGTCGTCTCGC

ori

8,240 8,250 8,260 8,270 8,280 8,290 8,300 8,310 8,320 8,330 8,340

CAGATACCAAATACTGTTCTTCTAGTGTAGCCGTAGTTAGGCCACCACTTCAAGAACTCTGTAGCACCGCCTACATACCTCGCTCTGCTAATCCTGTTACCAGTGGC
GTCTATGGTTTATGACAAGAAGATCACATCGGCATCAATCCGGTGGTGAAGTTCTTGAGACATCGTGGCGGATGTATGGAGCGAGACGATTAGGACAATGGTCACCG

ori

8,350 8,360 8,370 8,380 8,390 8,400 8,410 8,420 8,430 8,440 8,450

TGCTGCCAGTGGCGATAAGTCGTGTCTTACCGGGTTGGACTCAAGACGATAGTTACCGGATAAGGCGCAGCGGTCGGGCTGAACGGGGGGTTCGTGCACACAGCCCA
ACGACGGTCACCGCTATTCAGCACAGAATGGCCCAACCTGAGTTCTGCTATCAATGGCCTATTCCGCGTCGCCAGCCCGACTTGCCCCCCAAGCACGTGTGTCGGGT

ori

8,460 8,470 8,480 8,490 8,500 8,510 8,520 8,530 8,540 8,550 8,560

```
GCTTGGAGCGAACGACCTACACCGAACTGAGATACCTACAGCGTGAGCTATGAGAAAGCGCCACGCTTCCCGAAGGGAGAAAGGCGGACAGGTATCCGGTAAGCGGC
CGAACCTCGCTTGCTGGATGTGGCTTGACTCTATGGATGTCGCACTCGATACTCTTTCGCGGTGCGAAGGGCTTCCCTCTTTCCGCCTGTCCATAGGCCATTCGCCG
ori
8,570 8,580 8,590 8,600 8,610 8,620 8,630 8,640 8,650 8,660

AGGGTCGGAACAGGAGAGCGCACGAGGGAGCTTCCAGGGGGAAACGCCTGGTATCTTTATAGTCCTGTCGGGTTTCGCCACCTCTGACTTGAGCGTCGATTTTTGTG
TCCCAGCCTTGTCCTCTCGCGTGCTCCCTCGAAGGTCCCCCTTTGCGGACCATAGAAATATCAGGACAGCCCAAAGCGGTGGAGACTGAACTCGCAGCTAAAAACAC
ori
8,670 8,680 8,690 8,700 8,710 8,720 8,730 8,740 8,750 8,760 8,770

ATGCTCGTCAGGGGGGCGGAGCCTGTGGAAAAACGCCAGCAACGCGGCCTTTTTACGGTTCCTGGCCTTTTGCTGGCCTTTTGCTCACATGTTCTTTCCTGCGTTAT
TACGAGCAGTCCCCCCGCCTCGGACACCTTTTTGCGGTCGTTGCGCCGGAAAAATGCCAAGGACCGGAAAACGACCGGAAAACGAGTGTACAAGAAAGGACGCAATA
ori
8,780 8,790 8,800 8,810 8,820 8,830 8,840 8,850 8,860 8,870 8,880

CCCCTGATTCTGTGGATAACCGTATTACCGCCTTTGAGTGAGCTGATACCGCTCGCCGCAGCCGAACGACCGAGCGCAGCGAGTCAGTGAGCGAGGAAGCCTGCATA
GGGGACTAAGACACCTATTGGCATAATGGCGGAAACTCACTCGACTATGGCGAGCGGCGTCGGCTTGCTGGCTCGCGTCGCTCAGTCACTCGCTCCTTCGGACGTAT
8,890 8,900 8,910 8,920 8,930 8,940 8,950 8,960 8,970 8,980

NotI
ACGCGAAGTAATCTTTTCGGTTTTAAAGAAAAAGGGCAGGGTGGTGACACCTTGCCCTTTTTTGCCGGACTGCAGCGGCCGCTTCTAGaa
TGCGCTTCATTAGAAAAGCCAAAATTTCTTTTTCCCGTCCCACCACTGTGGAACGGGAAAAAACGGCCTGACGTCGCCGGCGAAGATCtt
his operon terminator   Bio...ix
8,990 9,000 9,010 9,020 9,030 9,040 9,050 9,060 9,070
```
