# Supplementary material for: Design of synthetic selenopeptides with antioxidant activity for the treatment of XP and non-melanoma skin cancer
Source: Front Syst Biol. 2026 Apr 9;6:1686085. doi: 10.3389/fsysb.2026.1686085 (PMC13102595; doi:10.3389/fsysb.2026.1686085)

pSB1C3 vector (2070 bp):

Source | Benchling link

Not I cut site: GCGGCCGC

Sequence:
TACTAGTAGCGGCCGCTGCAGTCCGGCAAAAAAGGGCAAGGTGTCACCACCCTGCCCT
TTTTCTTTAAAACCGAAAAGATTACTTCGCGTTATGCAGGCTTCCTCGCTCACTGACTCG
CTGCGCTCGGTCGTTCGGCTGCGGCGAGCGGTATCAGCTCACTCAAAGGCGGTAATAC
GGTTATCCACAGAATCAGGGGATAACGCAGGAAAGAACATGTGAGCAAAAGGCCAGCAA
AAGGCCAGGAACCGTAAAAAGGCCGCGTTGCTGGCGTTTTTCCACAGGCTCCGCCCCC
CTGACGAGCATCACAAAAATCGACGCTCAAGTCAGAGGTGGCGAAACCCGACAGGACT
ATAAAGATACCAGGCGTTTCCCCCTGGAAGCTCCCTCGTGCGCTCTCCTGTTCCGACCC
TGCCGCTTACCGGATACCTGTCCGCCTTTCTCCCTTCGGGAAGCGTGGCGCTTTCTCAT
AGCTCACGCTGTAGGTATCTCAGTTCGGTGTAGGTCGTTCGCTCCAAGCTGGGCTGTGT
GCACGAACCCCCCGTTCAGCCCGACCGCTGCGCCTTATCCGGTAACTATCGTCTTGAGT
CCAACCCGGTAAGACACGACTTATCGCCACTGGCAGCAGCCACTGGTAACAGGATTAGC
AGAGCGAGGTATGTAGGCGGTGCTACAGAGTTCTTGAAGTGGTGGCCTAACTACGGCTA
CACTAGAAGAACAGTATTTGGTATCTGCGCTCTGCTGAAGCCAGTTACCTTCGGAAAAAG
AGTTGGTAGCTCTTGATCCGGCAAACAAACCACCGCTGGTAGCGGTGGTTTTTTTGTTT
GCAAGCAGCAGATTACGCGCAGAAAAAAAGGATCTCAAGAAGATCCTTTGATCTTTTCTA
CGGGGTCTGACGCTCAGTGGAACGAAAACTCACGTTAAGGGATTTTGGTCATGAGATTA
TCAAAAAGGATCTTCACCTAGATCCTTTTAAATTAAAAATGAAGTTTTAAATCAATCTAAAG
TATATATGAGTAAACTTGGTCTGACAGCTCGAGGCTTGGATTCTCACCAATAAAAAACGC
CCGGCGGCAACCGAGCGTTCTGAACAAATCCAGATGGAGTTCTGAGGTCATTACTGGAT
CTATCAACAGGAGTCCAAGCGAGCTCGATATCAAATTACGCCCCGCCCTGCCACTCATC
GCAGTACTGTTGTAATTCATTAAGCATTCTGCCGACATGGAAGCCATCACAAACGGCATG
ATGAACCTGAATCGCCAGCGGCATCAGCACCTTGTCGCCTTGCGTATAATATTTGCCCAT
GGTGAAAACGGGGGCGAAGAAGTTGTCCATATTGGCCACGTTTAAATCAAAACTGGTGA
AACTCACCCAGGGATTGGCTGAGACGAAAAACATATTCTCAATAAACCCTTTAGGGAAAT
AGGCCAGGTTTTCACCGTAACACGCCACATCTTGCGAATATATGTGTAGAAACTGCCGGA
AATCGTCGTGGTATTCACTCCAGAGCGATGAAAACGTTTCAGTTTGCTCATGGAAAACG
GTGTAACAAGGGTGAACACTATCCCATATCACCAGCTCACCGTCTTTCATTGCCATACGA
AATTCCGGATGAGCATTCATCAGGCGGGCAAGAATGTGAATAAAGGCCGGATAAAACTT
GTGCTTATTTTTCTTTACGGTCTTTAAAAAGGCCGTAATATCCAGCTGAACGGTCTGGTTA
TAGGTACATTGAGCAACTGACTGAAATGCCTCAAAATGTTCTTTACGATGCCATTGGGATA
TATCAACGGTGGTATATCCAGTGATTTTTTTCTCCATTTTAGCTTCCTTAGCTCCTGAAAAT
CTCGATAACTCAAAAAATACGCCCGGTAGTGATCTTATTTCATTATGGTGAAAGTTGGAAC
CTCTTACGTGCCCGATCAACTCGAGTGCCACCTGACGTCTAAGAAACCATTATTATCATG
ACATTAACCTATAAAAATAGGCGTATCACGAGGCAGAATTTCAGATAAAAAAAATCCTTAG
CTTTCGCTAAGGATGATTTCTGGAATTCGCGGCCGCTTCTAGAG

lac promoter (55 bp):

Source | Benchling link
Sequence:
aattgtgagcggataacaattgacattgtgagcggataacaagatactgagcaca

RBS (12 bp):

Source | Benchling link

Sequence:
aaagaggagaaa

selA gene (1389 bp):

Source | Benchling link

Sequence:
atgaccaccgaaacccgcagcctgtatagccagctgccggcgattgatcgcctgctgcgcgatagcagctttctgagcctgcgc
gatacctatggccatacccgcgtggtggaactgctgcgccagatgctggatgaagcgcgcgaagtgattcgcggcagccagac
cctgccggcgtggtgcgaaaactgggcgcaggaagtggatgcgcgcctgaccaaagaagcgcagagcgcgctgcgcccgg
tgattaacctgaccggcaccgtgctgcataccaacctgggccgcgcgctgcaggcggaagcggcggtggaagcggtggcgc
aggcgatgcgcagcccggtgaccctggaatatgatctggatgatgcgggccgcggccatcgcgatcgcgcgctggcgcagct
gctgtgccgcattaccggcgcggaagatgcgtgcattgtgaacaacaacgcggcggcggtgctgctgatgctggcggcgaccg
cgagcggcaaagaagtggtggtgagccgcggcgaactggtggaaattggcggcgcgtttcgcattccggatgtgatgcgccag
gcgggctgcaccctgcatgaagtgggcaccaccaaccgcacccatgcgaacgattatcgccaggcggtgaacgaaaacacc
gcgctgctgatgaaagtgcataccagcaactatagcattcagggctttaccaaagcgattgatgaagcggaactggtggcgctg
ggcaaagaactggatgtgccggtggtgaccgatctgggcagcggcagcctggtggatctgagccagtatggcctgccgaaag
aaccgatgccgcaggaactgattgcggcgggcgtgagcctggtgagctttagcggcgataaactgctgggcggcccgcaggc
gggcattattgtgggcaaaaaagaaatgattgcgcgcctgcagagccatccgctgaaacgcgcgctgcgcgcggataaaatg
accctggcggcgctggaagcgaccctgcgcctgtatctgcatccggaagcgctgagcgaaaaactgccgaccctgcgcctgct
gacccgcagcgcggaagtgattcagattcaggcgcagcgcctgcaggcgccgctggcggcgcattatggcgcggaatttgcg
gtgcaggtgatgccgtgcctgagccagattggcagcggcagcctgccggtggatcgcctgccgagcgcggcgctgacctttacc
ccgcatgatggccgcggcagccatctggaaagcctggcggcgcgctggcgcgaactgccggtgccggtgattggccgcattta
tgatggccgcctgtggctggatctgcgctgcctggaagatgaacagcgctttctggaaatgctgctgaaa

selB gene (423 bp):

Source | Benchling link

Sequence:
MNNATAGHVDHGKTTANTGVNADRKKRGMTNDGYAYWDGRVGNDVGHKSNMAGVGGN
DHAVVACDDGVMATRHANTGNMTVATKADRVDARVDVRVKVRYGAAKNTAATGRGMDAR
HRHASHSRANDRATVKGAGVVTGTASGVKVGDSWTGVNKMRVRAHANTTANAGRNANNA
GDAKNNRGDWADVTRVNVTHTTWHNHHAASHVTGRVSDNAVDTWADNDRVRDNSARNT
AGARVVMNRRGKRKYWASARASDADASVHRGAVNADAWARNGGMRGYNAGYSNAVAAR
WRKNDTATYHHRDGGRRRRMAMDAVNKMRSGDNHSHHGWHDHKAGSANWKAGDWWV
RDAKTGTDAMRTRAAGNNTANVKDRYYRNDRNVANMNRDDCGSTCAADRDRGVGRKAN
NYDRNGTRRRGNDHRDAK

Selenocysteine-tRNA specific (EEFSEC) (2205 bp):

Source | Benchiling link

Sequence:
gcgggtgtccgaggcggcggcggcggcatggcagggcggcgggtgaacgtgaacgtgggcgtgctgggccacatcgacag
cggcaagacggcgctggcgcgggcgctaagcaccacagcctccaccgccgcctttgacaagcagccgcagagccgcgagc
gcggcatcacgctcgatctgggcttctcgtgcttctcggtgccgctgcccgcgcgcctgcggtcgtctttgcccgagttccaggcag
cgcccgaggccgagcccgagcccggcgagccactgcttcaggtcacgctggtcgactgccccgggcacgcctccctcatccg
gaccatcatcggcggggcccagatcattgatctgatgatgctggtcatcgatgtgaccaaggggatgcagacccagtcagcgg
aatgccttgtgatcggccagattgcctgccagaagctggtcgtggtgctgaacaaaatagacctcttacctgaaggaaagagac
aggcagcaattgataaaatgaccaagaaaatgcagaagaccctagagaacaccaagttccgaggtgcaccgattatacccgt
ggcggccaagccgggggggaccagaggcccccgaaactgaagctccacagggcattccagagctcattgagctcctgacgtc
ccagatttccatcccaacgagagatccctcgggaccgttcctcatgtctgtggaccactgtttctccatcaaaggccaaggcactgt
gatgacagggaccatcctttcaggctccatcagcctcggtgacagtgtggagatccctgccctcaaggtggtgaagaaggtgaa
gtccatgcagatgttccacatgcccatcacttcagccatgcaaggagaccggctgggcatctgcgtcacccagtttgaccctaag
ctgctggagcgcgggttggtgtgtgcccccgagtccctgcacactgtccatgcggccctcatctctgtggaaaagataccgtatttc
cgggggcccctgcaaaccaaggccaagttccacattacagtgggccatgaaacagtcatgggccggttgatgttcttcagtcctg
ctccagataactttgaccaggagcctatactggactctttcaacttctctcaagaataccttttccaggagcagtacctgtccaagga
tttgacaccagcagtgacagacaatgatgaggccgacaagaaggccggccaggccacagagggccattgtcctcggcagca
gtgggccctggtggagtttgagaagcccgtcacctgccctcggctgtgcctggtgattggctccaggctagatgcggacattcaca
ccaacacgtgccggctagccttccatggcatcctgctccacgggctagaggacaggaactacgccgacagcttcctgcccagg
ctgaaggtgtacaagctgaagcacaagcatggccttgtggagcgggcgatggatgactacagtgtgatcggccgctccctgttc
aaaaaggaaaccaacatccagctcttcgtggggctcaaggtgcacttgtccactggggaactgggcatcatcgacagtgccttc
ggccagagcggcaagttcaagatccacatcccaggtggcctcagccccgagtccaagaagatcctgacacccgccctcaag
aagcgggcccgggctggccgtggggaggccaccaggcaggaggagagcgccgagcggagcgagccctcacagcatgtg
gtgctcagcctgactttcaagcgttatgtcttcgacacccacaagcgcatggttcagtctccctgagtgtccggtgacctcccccag
ggcctccttgcccagcccagtccaggctgctgtgccaaatcccaaccagccacgcctcagcctctcccagtctctccctgcagtc
ctgcagcagcagcccccacccccaagcttggtgctgagccctggtgaggagctgaggggggatgggttgctggggccaggagg
gtctctcctccagcccctgcacactcccacccaggacagcccccagcccaactaggaaagggccatgggcagagggctggta
gccagtatcttccactgccccatctgttggccacctgcaggccagtctcaaccctcccccaggtgggcaggcacttgatggctaca
aataaatgtcccgtggccccagcccactcta

T7 RNA Polymerase (2652 bp)

Source | Benchling link

Sequence:
atgaacacgattaacatcgctaagaacgacttctctgacatcgaactggctgctatcccgttcaacactctggctgaccattacggt
gagcgtttagctcgcgaacagttggcccttgagcatgagtcttacgagatgggtgaagcacgcttccgcaagatgtttgagcgtca
acttaaagctggtgaggttgcggataacgctgccgccaagcctctcatcactaccctactccctaagatgattgcacgcatcaacg
actggtttgaggaagtgaaagctaagcgcggcaagcgcccgacagccttccagttcctgcaagaaatcaagccggaagccgt
agcgtacatcaccattaagaccactctggcttgcctaaccagtgctgacaatacaaccgttcaggctgtagcaagcgcaatcggt
cgggccattgaggacgaggctcgcttcggtcgtatccgtgaccttgaagctaagcacttcaagaaaaacgttgaggaacaactc
aacaagcgcgtagggcacgtctacaagaaagcatttatgcaagttgtcgaggctgacatgctctctaagggtctactcggtggcg
aggcgtggtcttcgtggcataaggaagactctattcatgtaggagtacgctgcatcgagatgctcattgagtcaaccggaatggtt
agcttacaccgccaaaatgctggcgtagtaggtcaagactctgagactatcgaactcgcacctgaatacgctgaggctatcgca
acccgtgcaggtgcgctggctggcatctctccgatgttccaaccttgcgtagttcctcctaagccgtggactggcattactggtggtg
gctattgggctaacggtcgtcgtcctctggcgctggtgcgtactcacagtaagaaagcactgatgcgctacgaagacgtttacatg
cctgaggtgtacaaagcgattaacattgcgcaaaacaccgcatggaaaatcaacaagaaagtcctagcggtcgccaacgtaa
tcaccaagtggaagcattgtccggtcgaggacatccctgcgattgagcgtgaagaactcccgatgaaaccggaagacatcgac

atgaatcctgaggctctcaccgcgtggaaacgtgctgccgctgctgtgtaccgcaaggacagggctcgcaagtctcgccgtatc
agccttgagttcatgcttgagcaagccaataagtttgctaaccataaggccatctggttcccttacaacatggactggcgcggtcgt
gtttacgccgtgtcaatgttcaacccgcaaggtaacgatatgaccaaaggactgcttacgctggcgaaaggtaaaccaatcggt
aaggaaggttactactggctgaaaatccacggtgcaaactgtgcgggtgtcgataaggttccgttccctgagcgcatcaagttcat
tgaggaaaaccacgagaacatcatggcttgcgctaagtctccactggagaacacttggtgggctgagcaagattctccgttctgc
ttccttgcgttctgctttgagtacgctggggtacagcaccacggcctgagctataactgctcccttccgctggcgtttgacgggtcttgc
tctggcatccagcacttctccgcgatgctccgagatgaggtaggtggtcgcgcggttaacttgcttcctagtgagaccgttcaggac
atctacgggattgttgctaagaaagtcaacgagattctacaagcagacgcaatcaatgggaccgataacgaagtagttaccgtg
accgatgagaacactggtgaaatctctgagaaagtcaagctgggcactaaggcactggctggtcaatggctggctcacggtgtt
actcgcagtgtgactaagcgttcagtcatgacgctggcttacgggtccaaagagttcggcttccgtcaacaagtgctggaagata
ccattcagccagctattgattccggcaagggtccgatgttcactcagccgaatcaggctgctggatacatggctaagctgatttggg
aatctgtgagcgtgacggtggtagctgcggttgaagcaatgaactggcttaagtctgctgctaagctgctggctgctgaggtcaaa
gataagaagactggagagattcttcgcaagcgttgcgctgtgcattgggtaactcctgatggtttccctgtgtggcaggaatacaa
gaagcctattcagacgcgcttgaacctgatgttcctcggtcagttccgcttacagcctaccattaacaccaacaaagatagcgag
attgatgcacacaaacaggagtctggtatcgctcctaactttgtacacagccaagacggtagccaccttcgtaagactgtagtgtg
ggcacacgagaagtacggaatcgaatcttttgcactgattcacgactccttcggtaccattccggctgacgctgcgaacctgttca
aagcagtgcgcgaaactatggttgacacatatgagtcttgtgatgtactggctgatttctacgaccagttcgctgaccagttgcacg
agtctcaattggacaaaatgccagcacttccggctaaaggtaacttgaacctccgtgacatcttagagtcggacttcgcgttcgcgt
aa

rpoC Terminator (83 bp)

Source | Benchling link

Sequence
gtaatcgttaatccgcaaataacgtaaaaacccgcttcggcgggttttttatggggggagtttagggaaagagcatttgtca

T7 promoter (18 bp)

Source | Benchling link

Sequence:
TAATACGACTCACTATAG

RBS (41 bp)

Source | Benchling link

Sequence
TTCGAAAATTAATACGACTCACTATAGGGAGACCACAACGG

Selera-2 gene optimized (84 bp)

Benchling link

Sequence:
atggccgattcggaacgcctgcatcatagcgcacacaagaaaggctgggcggcgtgcacgaacttttcacgttgtacccgcaa
a

T7 Terminator (48 bp)

Source | Benchling link

Sequence
ctagcataaccccttggggcctctaaacgggtcttgaggggttttttg

Cassette (7051 bp):

In this step, we joined the sequences and added the biobricks prefix and suffix. This enables subsequent cleavage with Not I for insertion into pSB1C3.

Biobrick prefix: 5'-GAATTCGCGGCCGCTTCTAG-3'
Biobrick suffix: 5'-TACTAGTAGCGGCCGCTGCAG-3'
Not I cut site: 5'-GCGGCCGC-3'

- Insert 1 (6839 bp) | Benchling link

Sequence
aattgtgagcggataacaattgacattgtgagcggataacaagatactgagcacaaaagaggagaaaatgaccaccgaaac
ccgcagcctgtatagccagctgccggcgattgatcgcctgctgcgcgatagcagctttctgagcctgcgcgatacctatggccata
cccgcgtggtggaactgctgcgccagatgctggatgaagcgcgcgaagtgattcgcggcagccagaccctgccggcgtggtg
cgaaaactgggcgcaggaagtggatgcgcgcctgaccaaagaagcgcagagcgcgctgcgcccggtgattaacctgaccg
gcaccgtgctgcataccaacctgggccgcgcgctgcaggcggaagcggcggtggaagcggtggcgcaggcgatgcgcagc
ccggtgaccctggaatatgatctggatgatgcgggccgcggccatcgcgatcgcgcgctggcgcagctgctgtgccgcattacc
ggcgcggaagatgcgtgcattgtgaacaacaacgcggcggcggtgctgctgatgctggcggcgaccgcgagcggcaaaga
agtggtggtgagccgcggcgaactggtggaaattggcggcgcgtttcgcattccggatgtgatgcgccaggcgggctgcaccct
gcatgaagtgggcaccaccaaccgcacccatgcgaacgattatcgccaggcggtgaacgaaaacaccgcgctgctgatgaa
agtgcataccagcaactatagcattcagggctttaccaaagcgattgatgaagcggaactggtggcgctgggcaaagaactgg
atgtgccggtggtgaccgatctgggcagcggcagcctggtggatctgagccagtatggcctgccgaaagaaccgatgccgcag
gaactgattgcggcgggcgtgagcctggtgagctttagcggcgataaactgctgggcggcccgcaggcgggcattattgtgggc
aaaaaagaaatgattgcgcgcctgcagagccatccgctgaaacgcgcgctgcgcgcggataaaatgaccctggcggcgctg
gaagcgaccctgcgcctgtatctgcatccggaagcgctgagcgaaaaactgccgaccctgcgcctgctgacccgcagcgcgg
aagtgattcagattcaggcgcagcgcctgcaggcgccgctggcggcgcattatggcgcggaatttgcggtgcaggtgatgccgt
gcctgagccagattggcagcggcagcctgccggtggatcgcctgccgagcgcggcgctgacctttaccccgcatgatggccgc
ggcagccatctggaaagcctggcggcgcgctggcgcgaactgccggtgccggtgattggccgcatttatgatggccgcctgtgg
ctggatctgcgctgcctggaagatgaacagcgctttctggaaatgctgctgaaaMNNATAGHVDHGKTTANTGVNA
DRKKRGMTNDGYAYWDGRVGNDVGHKSNMAGVGGNDHAVVACDDGVMATRHANTGNM
TVATKADRVDARVDVRVKVRYGAAKNTAATGRGMDARHRHASHSRANDRATVKGAGVVT
GTASGVKVGDSWTGVNKMRVRAHANTTANAGRNANNAGDAKNNRGDWADVTRVNVTHT
TWHNHHAASHVTGRVSDNAVDTWADNDRVRDNSARNTAGARVVMNRRGKRKYWASARA
SDADASVHRGAVNADAWARNGGMRGYNAGYSNAVAARWRKNDTATYHHRDGGRRRRMA
MDAVNKMRSGDNHSHHGWHDHKAGSANWKAGDWWVRDAKTGTDAMRTRAAGNNTAN
VKDRYYRNDRNVANMNRDDCGSTCAADRDRGVGRKANNYDRNGTRRRGNDHRDAKgcg
ggtgtccgaggcggcggcggcggcatggcagggcggcgggtgaacgtgaacgtgggcgtgctgggccacatcgacagcgg
caagacggcgctggcgcgggcgctaagcaccacagcctccaccgccgcctttgacaagcagccgcagagccgcgagcgcg
gcatcacgctcgatctgggcttctcgtgcttctcggtgccgctgcccgcgcgcctgcggtcgtctttgcccgagttccaggcagcgc
ccgaggccgagcccgagcccggcgagccactgcttcaggtcacgctggtcgactgccccgggcacgcctccctcatccggac

catcatcggcggggcccagatcattgatctgatgatgctggtcatcgatgtgaccaaggggatgcagacccagtcagcggaatg
ccttgtgatcggccagattgcctgccagaagctggtcgtggtgctgaacaaaatagacctcttacctgaaggaaagagacaggc
agcaattgataaaatgaccaagaaaatgcagaagaccctagagaacaccaagttccgaggtgcaccgattatacccgtggcg
gccaagccgggggaccagaggcccccgaaactgaagctccacagggcattccagagctcattgagctcctgacgtcccag
atttccatcccaacgagagatccctcgggaccgttcctcatgtctgtggaccactgtttctccatcaaaggccaaggcactgtgatg
acagggaccatcctttcaggctccatcagcctcggtgacagtgtggagatccctgccctcaaggtggtgaagaaggtgaagtcc
atgcagatgttccacatgcccatcacttcagccatgcaaggagaccggctgggcatctgcgtcacccagtttgaccctaagctgct
ggagcgcgggttggtgtgtgcccccgagtccctgcacactgtccatgcggccctcatctctgtggaaaagataccgtatttccggg
ggcccctgcaaaccaaggccaagttccacattacagtgggccatgaaacagtcatgggccggttgatgttcttcagtcctgctcc
agataactttgaccaggagcctatactggactctttcaacttctctcaagaataccttttccaggagcagtacctgtccaaggatttga
caccagcagtgacagacaatgatgaggccgacaagaaggccggccaggccacagagggccattgtcctcggcagcagtgg
gccctggtggagtttgagaagcccgtcacctgccctcggctgtgcctggtgattggctccaggctagatgcggacattcacacca
acacgtgccggctagccttccatggcatcctgctccacgggctagaggacaggaactacgccgacagcttcctgcccaggctg
aaggtgtacaagctgaagcacaagcatggccttgtggagcgggcgatggatgactacagtgtgatcggccgctccctgttcaaa
aaggaaaccaacatccagctcttcgtggggctcaaggtgcacttgtccactggggaactgggcatcatcgacagtgccttcggc
cagagcggcaagttcaagatccacatcccaggtggcctcagccccgagtccaagaagatcctgacacccgccctcaagaag
cgggcccgggctggccgtggggaggccaccaggcaggaggagagcgccgagcggagcgagccctcacagcatgtggtgc
tcagcctgactttcaagcgttatgtcttcgacacccacaagcgcatggttcagtctccctgagtgtccggtgacctcccccagggcc
tccttgcccagcccagtccaggctgctgtgccaaatcccaaccagccacgcctcagcctctcccagtctctccctgcagtcctgca
gcagcagcccccacccccaagcttggtgctgagccctggtgaggagctgaggggggatgggttgctggggccaggagggtctct
cctccagcccctgcacactcccacccaggacagcccccagcccaactaggaaagggccatgggcagaggctggtagcca
gtatcttccactgccccatctgttggccacctgcaggccagtctcaaccctcccccaggtgggcaggcacttgatggctacaaata
aatgtcccgtggccccagcccactctaatgaacacgattaacatcgctaagaacgacttctctgacatcgaactggctgctatccc
gttcaacactctggctgaccattacggtgagcgtttagctcgcgaacagttggcccttgagcatgagtcttacgagatgggtgaag
cacgcttccgcaagatgtttgagcgtcaacttaaagctggtgaggttgcggataacgctgccgccaagcctctcatcactaccta
ctccctaagatgattgcacgcatcaacgactggtttgaggaagtgaaagctaagcgcggcaagcgcccgacagccttccagttc
ctgcaagaaatcaagccggaagccgtagcgtacatcaccattaagaccactctggcttgcctaaccagtgctgacaatacaac
cgttcaggctgtagcaagcgcaatcggtcgggccattgaggacgaggctcgcttcggtcgtatccgtgaccttgaagctaagcac
ttcaagaaaaacgttgaggaacaactcaacaagcgcgtagggcacgtctacaagaaagcatttatgcaagttgtcgaggctga
catgctctctaagggtctactcggtggcgaggcgtggtcttcgtggcataaggaagactctattcatgtaggagtacgctgcatcga
gatgctcattgagtcaaccggaatggttagcttacaccgccaaaatgctggcgtagtaggtcaagactctgagactatcgaactc
gcacctgaatacgctgaggctatcgcaacccgtgcaggtgcgctggctggcatctctccgatgttccaaccttgcgtagttcctcct
aagccgtggactggcattactggtggtggctattgggctaacggtcgtcgtcctctggcgctggtgcgtactcacagtaagaaagc
actgatgcgctacgaagacgtttacatgcctgaggtgtacaaagcgattaacattgcgcaaaacaccgcatggaaaatcaaca
agaaagtcctagcggtcgccaacgtaatcaccaagtggaagcattgtccggtcgaggacatccctgcgattgagcgtgaagaa
ctcccgatgaaaccggaagacatcgacatgaatcctgaggctctcaccgcgtggaaacgtgctgccgctgctgtgtaccgcaa
ggacagggctcgcaagtctcgccgtatcagccttgagttcatgcttgagcaagccaataagtttgctaaccataaggccatctggtt
cccttacaacatggactggcgcggtcgtgtttacgccgtgtcaatgttcaacccgcaaggtaacgatatgaccaaaggactgctta
cgctggcgaaaggtaaaccaatcggtaaggaaggttactactggctgaaaatccacggtgcaaactgtgcgggtgtcgataag
gttccgttccctgagcgcatcaagttcattgaggaaaaccacgagaacatcatggcttgcgctaagtctccactggagaacacttg
gtgggctgagcaagattctccgttctgcttccttgcgttctgctttgagtacgctggggtacagcaccacggcctgagctataactgct
cccttccgctggcgtttgacgggtcttgctctggcatccagcacttctccgcgatgctccgagatgaggtaggtggtcgcgcggtta
acttgcttcctagtgagaccgttcaggacatctacgggattgttgctaagaaagtcaacgagattctacaagcagacgcaatcaat
gggaccgataacgaagtagttaccgtgaccgatgagaacactggtgaaatctctgagaaagtcaagctgggcactaaggcact
ggctggtcaatggctggctcacggtgttactcgcagtgtgactaagcgttcagtcatgacgctggcttacgggtccaaagagttcg
gcttccgtcaacaagtgctggaagataccattcagccagctattgattccggcaagggtccgatgttcactcagccgaatcaggct
gctggatacatggctaagctgatttgggaatctgtgagcgtgacggtggtagctgcggttgaagcaatgaactggcttaagtctgct
gctaagctgctggctgctgaggtcaaagataagaagactggagagattcttcgcaagcgttgcgctgtgcattgggtaactcctga

tggtttccctgtgtggcaggaatacaagaagcctattcagacgcgcttgaacctgatgttcctcggtcagttccgcttacagcctacc
attaacaccaacaaagatagcgagattgatgcacacaaacaggagtctggtatcgctcctaactttgtacacagccaagacggt
agccaccttcgtaagactgtagtgtgggcacacgagaagtacggaatcgaatcttttgcactgattcacgactccttcggtaccatt
ccggctgacgctgcgaacctgttcaaagcagtgcgcgaaactatggttgacacatatgagtcttgtgatgtactggctgatttctac
gaccagttcgctgaccagttgcacgagtctcaattggacaaaatgccagcacttccggctaaaggtaacttgaacctccgtgaca
tcttagagtcggacttcgcgttcgcgtaagtaatcgttaatccgcaaataacgtaaaaacccgcttcggcgggtttttttatggggg
agtttagggaaagagcatttgtca

- Insert 2 (212 bp) | Benchling link

Sequence
TAATACGACTCACTATAGTTCGAAAATTAATACGACTCACTATAGGGAGACCACAACGGatg
gccgattcggaacgcctgcatcatagcgcacacaagaaaggctgggcggcgtgcacgaacttttcacgttgtacccgcaaact
agcataaccccttggggcctctaaacgggtcttgaggggttttttgTACTAGTAGCGGCCGCTGCAG

- Cassette (insert 1 and 2) (7051 bp) | Benchling link

GAATTCGCGGCCGCTTCTAGaattgtgagcggataacaattgacattgtgagcggataacaagatactgagcaca
aaagaggagaaaatgaccaccgaaacccgcagcctgtatagccagctgccggcgattgatcgcctgctgcgcgatagcagct
ttctgagcctgcgcgatacctatggccatacccgcgtggtggaactgctgcgccagatgctggatgaagcgcgcgaagtgattcg
cggcagccagaccctgccggcgtggtgcgaaaactgggcgcaggaagtggatgcgcgcctgaccaaagaagcgcagagc
gcgctgcgcccggtgattaacctgaccggcaccgtgctgcataccaacctgggccgcgcgctgcaggcggaagcggcggtgg
aagcggtggcgcaggcgatgcgcagcccggtgaccctggaatatgatctggatgatgcgggccgcggccatcgcgatcgcgc
gctggcgcagctgctgtgccgcattaccggcgcggaagatgcgtgcattgtgaacaacaacgcggcggcggtgctgctgatgct
ggcggcgaccgcgagcggcaaagaagtggtggtgagccgcggcgaactggtggaaattggcggcgcgtttcgcattccggat
gtgatgcgccaggcgggctgcaccctgcatgaagtgggcaccaccaaccgcacccatgcgaacgattatcgccaggcggtga
acgaaaacaccgcgctgctgatgaaagtgcataccagcaactatagcattcagggctttaccaaagcgattgatgaagcggaa
ctggtggcgctgggcaaagaactggatgtgccggtggtgaccgatctgggcagcggcagcctggtggatctgagccagtatgg
cctgccgaaagaaccgatgccgcaggaactgattgcggcgggcgtgagcctggtgagctttagcggcgataaactgctgggc
ggcccgcaggcgggcattattgtgggcaaaaaagaaatgattgcgcgcctgcagagccatccgctgaaacgcgcgctgcgcg
cggataaaatgaccctggcggcgctggaagcgaccctgcgcctgtatctgcatccggaagcgctgagcgaaaaactgccgac
cctgcgcctgctgacccgcagcgcggaagtgattcagattcaggcgcagcgcctgcaggcgccgctggcggcgcattatggcg
cggaatttgcggtgcaggtgatgccgtgcctgagccagattggcagcggcagcctgccggtggatcgcctgccgagcgcggcg
ctgacctttaccccgcatgatggccgcggcagccatctggaaagcctggcggcgcgctggcgcgaactgccggtgccggtgatt
ggccgcatttatgatggccgcctgtggctggatctgcgctgcctggaagatgaacagcgctttctggaaatgctgctgaaaMNN
ATAGHVDHGKTTANTGVNADRKKRGMTNDGYAYWDGRVGNDVGHKSNMAGVGGNDHAV
VACDDGVMATRHANTGNMTVATKADRVDARVDVRVKVRYGAAKNTAATGRGMDARHRHA
SHSRANDRATVKGAGVVTGTASGVKVGDSWTGVNKMRVRAHANTTANAGRNANNAGDAK
NNRGDWADVTRVNVTHTTWHNHHAASHVTGRVSDNAVDTWADNDRVRDNSARNTAGAR
VVMNRRGKRKYWASARASDADASVHRGAVNADAWARNGGMRGYNAGYSNAVAARWRK
NDTATYHHRDGGRRRRMAMDAVNKMRSGDNHSHHGWHDHKAGSANWKAGDWWVRDA
KTGTDAMRTRAAGNNTANVKDRYYRNDRNVANMNRDDCGSTCAADRDRGVGRKANNYD
RNGTRRRGNDHRDAKgcgggtgtccgaggcggcggcggcggcatggcagggcggcgggtgaacgtgaacgtgg
gcgtgctgggccacatcgacagcggcaagacggcgctggcgcgggcgctaagcaccacagcctccaccgccgcctttgaca
agcagccgcagagccgcgagcgcggcatcacgctcgatctgggcttctcgtgcttctcggtgccgctgcccgcgcgcctgcggt
cgtctttgcccgagttccaggcagcgcccgaggccgagcccgagcccggcgagccactgcttcaggtcacgctggtcgactgc
cccgggcacgcctccctcatccggaccatcatcggcggggcccagatcattgatctgatgatgctggtcatcgatgtgaccaagg
ggatgcagacccagtcagcggaatgccttgtgatcggccagattgcctgccagaagctggtcgtggtgctgaacaaaatagac

ctcttacctgaaggaaagagacaggcagcaattgataaaatgaccaagaaaatgcagaagaccctagagaacaccaagttc
cgaggtgcaccgattatacccgtggcggccaagccgggggaccagaggcccccgaaactgaagctccacagggcattcca
gagctcattgagctcctgacgtcccagatttccatcccaacgagagatccctcgggaccgttcctcatgtctgtggaccactgtttct
ccatcaaaggccaaggcactgtgatgacagggaccatcctttcaggctccatcagcctcggtgacagtgtggagatccctgccct
caaggtggtgaagaaggtgaagtccatgcagatgttccacatgcccatcacttcagccatgcaaggagaccggctgggcatct
gcgtcacccagtttgaccctaagctgctggagcgcgggttggtgtgtgcccccgagtccctgcacactgtccatgcggccctcatc
tctgtggaaaagataccgtatttccgggggcccctgcaaaccaaggccaagttccacattacagtgggccatgaaacagtcatg
ggccggttgatgttcttcagtcctgctccagataactttgaccaggagcctatactggactctttcaacttctctcaagaataccttttcc
aggagcagtacctgtccaaggatttgacaccagcagtgacagacaatgatgaggccgacaagaaggccggccaggccaca
gagggccattgtcctcggcagcagtgggccctggtggagtttgagaagcccgtcacctgccctcggctgtgcctggtgattggctc
caggctagatgcggacattcacaccaacacgtgccggctagccttccatggcatcctgctccacgggctagaggacaggaact
acgccgacagcttcctgcccaggctgaaggtgtacaagctgaagcacaagcatggccttgtggagcgggcgatggatgactac
agtgtgatcggccgctccctgttcaaaaaggaaaccaacatccagctcttcgtggggctcaaggtgcacttgtccactggggaac
tgggcatcatcgacagtgccttcggccagagcggcaagttcaagatccacatcccaggtggcctcagccccgagtccaagaag
atcctgacacccgccctcaagaagcgggcccgggctggccgtggggaggccaccaggcaggaggagagcgccgagcgg
agcgagccctcacagcatgtggtgctcagcctgactttcaagcgttatgtcttcgacacccacaagcgcatggttcagtctccctga
gtgtccggtgacctcccccagggcctccttgcccagcccagtccaggctgctgtgccaaatcccaaccagccacgcctcagcct
ctcccagtctctccctgcagtcctgcagcagcagcccccacccccaagcttggtgctgagccctggtgaggagctgagggggat
gggttgctggggccaggagggtctctcctccagcccctgcacactcccacccaggacagcccccagcccaactaggaaaggg
ccatgggcagagggctggtagccagtatcttccactgccccatctgttggccacctgcaggccagtctcaaccctcccccaggtg
ggcaggcacttgatggctacaaataaatgtcccgtggccccagcccactctaatgaacacgattaacatcgctaagaacgactt
ctctgacatcgaactggctgctatcccgttcaacactctggctgaccattacggtgagcgtttagctcgcgaacagttggcccttga
gcatgagtcttacgagatgggtgaagcacgcttccgcaagatgtttgagcgtcaacttaaagctggtgaggttgcggataacgct
gccgccaagcctctcatcactaccctactccctaagatgattgcacgcatcaacgactggtttgaggaagtgaaagctaagcgc
ggcaagcgcccgacagccttccagttcctgcaagaaatcaagccggaagccgtagcgtacatcaccattaagaccactctggc
ttgcctaaccagtgctgacaatacaaccgttcaggctgtagcaagcgcaatcggtcgggccattgaggacgaggctcgcttcggt
cgtatccgtgaccttgaagctaagcacttcaagaaaaacgttgaggaacaactcaacaagcgcgtagggcacgtctacaaga
aagcatttatgcaagttgtcgaggctgacatgctctctaagggtctactcggtggcgaggcgtggtcttcgtggcataaggaagact
ctattcatgtaggagtacgctgcatcgagatgctcattgagtcaaccggaatggttagcttacaccgccaaaatgctggcgtagta
ggtcaagactctgagactatcgaactcgcacctgaatacgctgaggctatcgcaacccgtgcaggtgcgctggctggcatctctc
cgatgttccaaccttgcgtagttcctcctaagccgtggactggcattactggtggtggctattgggctaacggtcgtcgtcctctggcg
ctggtgcgtactcacagtaagaaagcactgatgcgctacgaagacgtttacatgcctgaggtgtacaaagcgattaacattgcgc
aaaacaccgcatggaaaatcaacaagaaagtcctagcggtcgccaacgtaatcaccaagtggaagcattgtccggtcgagg
acatccctgcgattgagcgtgaagaactcccgatgaaaccggaagacatcgacatgaatcctgaggctctcaccgcgtggaaa
cgtgctgccgctgctgtgtaccgcaaggacagggctcgcaagtctcgccgtatcagccttgagttcatgcttgagcaagccaata
agtttgctaaccataaggccatctggttcccttacaacatggactggcgcggtcgtgtttacgccgtgtcaatgttcaacccgcaag
gtaacgatatgaccaaaggactgcttacgctggcgaaaggtaaaccaatcggtaaggaaggttactactggctgaaaatccac
ggtgcaaactgtgcgggtgtcgataaggttccgttccctgagcgcatcaagttcattgaggaaaaccacgagaacatcatggctt
gcgctaagtctccactggagaacacttggtgggctgagcaagattctccgttctgcttccttgcgttctgctttgagtacgctggggta
cagcaccacggcctgagctataactgctcccttccgctggcgtttgacgggtcttgctctggcatccagcacttctccgcgatgctcc
gagatgaggtaggtggtcgcgcggttaacttgcttcctagtgagaccgttcaggacatctacgggattgttgctaagaaagtcaac
gagattctacaagcagacgcaatcaatgggaccgataacgaagtagttaccgtgaccgatgagaacactggtgaaatctctga
gaaagtcaagctgggcactaaggcactggctggtcaatggctggctcacggtgttactcgcagtgtgactaagcgttcagtcatg
acgctggcttacgggtccaaagagttcggcttccgtcaacaagtgctggaagataccattcagccagctattgattccggcaagg
gtccgatgttcactcagccgaatcaggctgctggatacatggctaagctgatttgggaatctgtgagcgtgacggtggtagctgcg
gttgaagcaatgaactggcttaagtctgctgctaagctgctggctgctgaggtcaaagataagaagactggagagattcttcgca
agcgttgcgctgtgcattgggtaactcctgatggtttccctgtgtggcaggaatacaagaagcctattcagacgcgcttgaacctga
tgttcctcggtcagttccgcttacagcctaccattaacaccaacaaagatagcgagattgatgcacacaaacaggagtctggtatc

gctcctaactttgtacacagccaagacggtagccaccttcgtaagactgtagtgtgggcacacgagaagtacggaatcgaatctt
ttgcactgattcacgactccttcggtaccattccggctgacgctgcgaacctgttcaaagcagtgcgcgaaactatggttgacacat
atgagtcttgtgatgtactggctgatttctacgaccagttcgctgaccagttgcacgagtctcaattggacaaaatgccagcacttcc
ggctaaaggtaacttgaacctccgtgacatcttagagtcggacttcgcgttcgcgtaagtaatcgttaatccgcaaataacgtaaa
aacccgcttcggcgggtttttttatgggggggagtttagggaaagagcatttgtcaTAATACGACTCACTATAGTTCGA
AAATTAATACGACTCACTATAGGGAGACCACAACGGatggccgattcggaacgcctgcatcatagcgcac
acaagaaaggctgggcggcgtgcacgaacttttcacgttgtacccgcaaactagcataaccccttggggcctctaaacgggtctt
gaggggttttttgTACTAGTAGCGGCCGCTGCAG

pSec-Reg vector (9078 bp):

Benchling link

Sequence
ttgtgagcggataacaattgacattgtgagcggataacaagatactgagcacaaaagaggagaaaatgaccaccgaaaccc
gcagcctgtatagccagctgccggcgattgatcgcctgctgcgcgatagcagctttctgagcctgcgcgatacctatggccatacc
cgcgtggtggaactgctgcgccagatgctggatgaagcgcgcgaagtgattcgcggcagccagaccctgccggcgtggtgcg
aaaactgggcgcaggaagtggatgcgcgcctgaccaaagaagcgcagagcgcgctgcgcccggtgattaacctgaccggc
accgtgctgcataccaacctgggccgcgcgctgcaggcggaagcggcggtggaagcggtggcgcaggcgatgcgcagccc
ggtgaccctggaatatgatctggatgatgcgggccgcggccatcgcgatcgcgcgctggcgcagctgctgtgccgcattaccgg
cgcggaagatgcgtgcattgtgaacaacaacgcggcggcggtgctgctgatgctggcggcgaccgcgagcggcaaagaagt
ggtggtgagccgcggcgaactggtggaaattggcggcgcgtttcgcattccggatgtgatgcgccaggcgggctgcaccctgca
tgaagtgggcaccaccaaccgcacccatgcgaacgattatcgccaggcggtgaacgaaaacaccgcgctgctgatgaaagt
gcataccagcaactatagcattcagggctttaccaaagcgattgatgaagcggaactggtggcgctgggcaaagaactggatgt
gccggtggtgaccgatctgggcagcggcagcctggtggatctgagccagtatggcctgccgaaagaaccgatgccgcaggaa
ctgattgcggcgggcgtgagcctggtgagctttagcggcgataaactgctgggcggcccgcaggcgggcattattgtgggcaaa
aaagaaatgattgcgcgcctgcagagccatccgctgaaacgcgcgctgcgcgcggataaaatgaccctggcggcgctggaa
gcgaccctgcgcctgtatctgcatccggaagcgctgagcgaaaaactgccgaccctgcgcctgctgacccgcagcgcggaag
tgattcagattcaggcgcagcgcctgcaggcgccgctggcggcgcattatggcgcggaatttgcggtgcaggtgatgccgtgcct
gagccagattggcagcggcagcctgccggtggatcgcctgccgagcgcggcgctgacctttaccccgcatgatggccgcggc
agccatctggaaagcctggcggcgcgctggcgcgaactgccggtgccggtgattggccgcatttatgatggccgcctgtggctg
gatctgcgctgcctggaagatgaacagcgctttctggaaatgctgctgaaaMNNATAGHVDHGKTTANTGVNADR
KKRGMTNDGYAYWDGRVGNDVGHKSNMAGVGGNDHAVVACDDGVMATRHANTGNMTV
ATKADRVDARVDVRVKVRYGAAKNTAATGRGMDARHRHASHSRANDRATVKGAGVVTGT
ASGVKVGDSWTGVNKMRVRAHANTTANAGRNANNAGDAKNNRGDWADVTRVNVTHTTW
HNHHAASHVTGRVSDNAVDTWADNDRVRDNSARNTAGARVVMNRRGKRKYWASARASD
ADASVHRGAVNADAWARNGGMRGYNAGYSNAVAARWRKNDTATYHHRDGGRRRRMAM
DAVNKMRSGDNHSHHGWHDHKAGSANWKAGDWWVRDAKTGTDAMRTRAAGNNTANVK
DRYYRNDRNVANMNRDDCGSTCAADRDRGVGRKANNYDRNGTRRRGNDHRDAKgcgggt
gtccgaggcggcggcggcggcatggcagggcggcgggtgaacgtgaacgtgggcgtgctgggccacatcgacagcggca
agacggcgctggcgcgggcgctaagcaccacagcctccaccgccgcctttgacaagcagccgcagagccgcgagcgcggc
atcacgctcgatctgggcttctcgtgcttctcggtgccgctgcccgcgcgcctgcggtcgtctttgcccgagttccaggcagcgccc
gaggccgagcccgagcccggcgagccactgcttcaggtcacgctggtcgactgccccgggcacgcctccctcatccggacca
tcatcggcggggcccagatcattgatctgatgatgctggtcatcgatgtgaccaaggggatgcagacccagtcagcggaatgcc
ttgtgatcggccagattgcctgccagaagctggtcgtggtgctgaacaaaatagacctcttacctgaaggaaagagacaggcag
caattgataaaatgaccaagaaaatgcagaagaccctagagaacaccaagttccgaggtgcaccgattatacccgtggcggc
caagccgggggggaccagaggcccccgaaactgaagctccacagggcattccagagctcattgagctcctgacgtcccagattt
ccatcccaacgagagatccctcgggaccgttcctcatgtctgtggaccactgtttctccatcaaaggccaaggcactgtgatgaca

gggaccatcctttcaggctccatcagcctcggtgacagtgtggagatccctgccctcaaggtggtgaagaaggtgaagtccatgc
agatgttccacatgcccatcacttcagccatgcaaggagaccggctgggcatctgcgtcacccagtttgaccctaagctgctgga
gcgcgggttggtgtgtgcccccgagtccctgcacactgtccatgcggccctcatctctgtggaaaagataccgtatttccgggggc
ccctgcaaaccaaggccaagttccacattacagtgggccatgaaacagtcatgggccggttgatgttcttcagtcctgctccagat
aactttgaccaggagcctatactggactctttcaacttctctcaagaatacctttccaggagcagtacctgtccaaggatttgacac
cagcagtgacagacaatgatgaggccgacaagaaggccggccaggccacagagggccattgtcctcggcagcagtgggcc
ctggtggagtttgagaagcccgtcacctgccctcggctgtgcctggtgattggctccaggctagatgcggacattcacaccaaca
cgtgccggctagccttccatggcatcctgctccacgggctagaggacaggaactacgccgacagcttcctgcccaggctgaag
gtgtacaagctgaagcacaagcatggccttgtggagcgggcgatggatgactacagtgtgatcggccgctccctgttcaaaaag
gaaaccaacatccagctcttcgtggggctcaaggtgcacttgtccactggggaactgggcatcatcgacagtgccttcggccag
agcggcaagttcaagatccacatcccaggtggcctcagccccgagtccaagaagatcctgacacccgccctcaagaagcgg
gcccgggctggccgtggggaggccaccaggcaggaggagagcgccgagcggagcgagccctcacagcatgtggtgctca
gcctgactttcaagcgttatgtcttcgacacccacaagcgcatggttcagtctccctgagtgtccggtgacctcccccagggcctcct
tgcccagcccagtccaggctgctgtgccaaatcccaaccagccacgcctcagcctctcccagtctctccctgcagtcctgcagca
gcagcccccacccccaagcttggtgctgagccctggtgaggagctgaggggggatgggttgctggggccaggagggtctctcct
ccagcccctgcacactcccacccaggacagcccccagcccaactaggaaagggccatgggcagagggctggtagccagta
tcttccactgccccatctgttggccacctgcaggccagtctcaaccctcccccaggtgggcaggcacttgatggctacaaataaat
gtcccgtggccccagcccactctaatgaacacgattaacatcgctaagaacgacttctctgacatcgaactggctgctatcccgtt
caacactctggctgaccattacggtgagcgtttagctcgcgaacagttggcccttgagcatgagtcttacgagatgggtgaagca
cgcttccgcaagatgtttgagcgtcaacttaaagctggtgaggttgcggataacgctgccgccaagcctctcatcactaccctactc
cctaagatgattgcacgcatcaacgactggtttgaggaagtgaaagctaagcgcggcaagcgcccgacagccttccagttcctg
caagaaatcaagccggaagccgtagcgtacatcaccattaagaccactctggcttgcctaaccagtgctgacaatacaaccgtt
caggctgtagcaagcgcaatcggtcgggccattgaggacgaggctcgcttcggtcgtatccgtgaccttgaagctaagcacttca
agaaaaacgttgaggaacaactcaacaagcgcgtagggcacgtctacaagaaagcatttatgcaagttgtcgaggctgacat
gctctctaagggtctactcggtggcgaggcgtggtcttcgtggcataaggaagactctattcatgtaggagtacgctgcatcgagat
gctcattgagtcaaccggaatggttagcttacaccgccaaaatgctggcgtagtaggtcaagactctgagactatcgaactcgca
cctgaatacgctgaggctatcgcaacccgtgcaggtgcgctggctggcatctctccgatgttccaaccttgcgtagttcctcctaag
ccgtggactggcattactggtggtggctattgggctaacggtcgtcgtcctctggcgctggtgcgtactcacagtaagaaagcact
gatgcgctacgaagacgtttacatgcctgaggtgtacaaagcgattaacattgcgcaaaacaccgcatggaaaatcaacaaga
aagtcctagcggtcgccaacgtaatcaccaagtggaagcattgtccggtcgaggacatccctgcgattgagcgtgaagaactcc
cgatgaaaccggaagacatcgacatgaatcctgaggctctcaccgcgtggaaacgtgctgccgctgctgtgtaccgcaaggac
agggctcgcaagtctcgccgtatcagccttgagttcatgcttgagcaagccaataagtttgctaaccataaggccatctggttccctt
acaacatggactggcgcggtcgtgtttacgccgtgtcaatgttcaacccgcaaggtaacgatatgaccaaaggactgcttacgct
ggcgaaaggtaaaccaatcggtaaggaaggttactactggctgaaaatccacggtgcaaactgtgcgggtgtcgataaggttc
cgttccctgagcgcatcaagttcattgaggaaaaccacgagaacatcatggcttgcgctaagtctccactggagaacacttggtg
ggctgagcaagattctccgttctgcttccttgcgttctgctttgagtacgctggggtacagcaccacggcctgagctataactgctccc
ttccgctggcgtttgacgggtcttgctctggcatccagcacttctccgcgatgctccgagatgaggtaggtggtcgcgcggttaactt
gcttcctagtgagaccgttcaggacatctacgggattgttgctaagaaagtcaacgagattctacaagcagacgcaatcaatggg
accgataacgaagtagttaccgtgaccgatgagaacactggtgaaatctctgagaaagtcaagctgggcactaaggcactggc
tggtcaatggctggctcacggtgttactcgcagtgtgactaagcgttcagtcatgacgctggcttacgggtccaaagagttcggctt
ccgtcaacaagtgctggaagataccattcagccagctattgattccggcaagggtccgatgttcactcagccgaatcaggctgct
ggatacatggctaagctgatttgggaatctgtgagcgtgacggtggtagctgcggttgaagcaatgaactggcttaagtctgctgct
aagctgctggctgctgaggtcaaagataagaagactggagagattcttcgcaagcgttgcgctgtgcattgggtaactcctgatg
gtttccctgtgtggcaggaatacaagaagcctattcagacgcgcttgaacctgatgttcctcggtcagttccgcttacagcctaccat
taacaccaacaaagatagcgagattgatgcacacaaacaggagtctggtatcgctcctaactttgtacacagccaagacggta
gccaccttcgtaagactgtagtgtgggcacacgagaagtacggaatcgaatcttttgcactgattcacgactccttcggtaccattc
cggctgacgctgcgaacctgttcaaagcagtgcgcgaaactatggttgacacatatgagtcttgtgatgtactggctgatttctacg
accagttcgctgaccagttgcacgagtctcaattggacaaaatgccagcacttccggctaaaggtaacttgaacctccgtgacat

cttagagtcggacttcgcgttcgcgtaagtaatcgttaatccgcaaataacgtaaaaacccgcttcggcgggtttttttatgggggga
gtttagggaaagagcatttgtcaTAATACGACTCACTATAGTTCGAAAATTAATACGACTCACTATAGG
GAGACCACAACGGatggccgattcggaacgcctgcatcatagcgcacacaagaaaggctgggcggcgtgcacgaa
cttttcacgttgtacccgcaaactagcataaccccttggggcctctaaacgggtcttgaggggtttttttgTACTAGTAGCGGC
CGCGAATTCCAGAAATCATCCTTAGCGAAAGCTAAGGATTTTTTTTATCTGAAATTCTGCC
TCGTGATACGCCTATTTTTATAGGTTAATGTCATGATAATAATGGTTTCTTAGACGTCAGGT
GGCACTCGAGTTGATCGGGCACGTAAGAGGTTCCAACTTTCACCATAATGAAATAAGATC
ACTACCGGGCGTATTTTTTGAGTTATCGAGATTTTCAGGAGCTAAGGAAGCTAAAATGGA
GAAAAAAATCACTGGATATACCACCGTTGATATATCCCAATGGCATCGTAAAGAACATTTT
GAGGCATTTCAGTCAGTTGCTCAATGTACCTATAACCAGACCGTTCAGCTGGATATTACG
GCCTTTTTAAAGACCGTAAAGAAAAATAAGCACAAGTTTTATCCGGCCTTTATTCACATTC
TTGCCCGCCTGATGAATGCTCATCCGGAATTTCGTATGGCAATGAAAGACGGTGAGCTG
GTGATATGGGATAGTGTTCACCCTTGTTACACCGTTTTCCATGAGCAAACTGAAACGTTT
TCATCGCTCTGGAGTGAATACCACGACGATTTCCGGCAGTTTCTACACATATATTCGCAA
GATGTGGCGTGTTACGGTGAAAACCTGGCCTATTTCCCTAAAGGGTTTATTGAGAATATG
TTTTTCGTCTCAGCCAATCCCTGGGTGAGTTTCACCAGTTTTGATTTAAACGTGGCCAAT
ATGGACAACTTCTTCGCCCCCGTTTTCACCATGGGCAAATATTATACGCAAGGCGACAAG
GTGCTGATGCCGCTGGCGATTCAGGTTCATCATGCCGTTTGTGATGGCTTCCATGTCGG
CAGAATGCTTAATGAATTACAACAGTACTGCGATGAGTGGCAGGGCGGGGCGTAATTTG
ATATCGAGCTCGCTTGGACTCCTGTTGATAGATCCAGTAATGACCTCAGAACTCCATCTG
GATTTGTTCAGAACGCTCGGTTGCCGCCGGGCGTTTTTTATTGGTGAGAATCCAAGCCT
CGAGCTGTCAGACCAAGTTTACTCATATATACTTTAGATTGATTTAAAACTTCATTTTTAATT
TAAAAGGATCTAGGTGAAGATCCTTTTTGATAATCTCATGACCAAAATCCCTTAACGTGAG
TTTTCGTTCCACTGAGCGTCAGACCCCGTAGAAAAGATCAAAGGATCTTCTTGAGATCCT
TTTTTTCTGCGCGTAATCTGCTGCTTGCAAACAAAAAAACCACCGCTACCAGCGGTGGT
TTGTTTGCCGGATCAAGAGCTACCAACTCTTTTTCCGAAGGTAACTGGCTTCAGCAGAG
CGCAGATACCAAATACTGTTCTTCTAGTGTAGCCGTAGTTAGGCCACCACTTCAAGAACT
CTGTAGCACCGCCTACATACCTCGCTCTGCTAATCCTGTTACCAGTGGCTGCTGCCAGT
GGCGATAAGTCGTGTCTTACCGGGTTGGACTCAAGACGATAGTTACCGGATAAGGCGCA
GCGGTCGGGCTGAACGGGGGGTTCGTGCACACAGCCCAGCTTGGAGCGAACGACCTA
CACCGAACTGAGATACCTACAGCGTGAGCTATGAGAAAGCGCCACGCTTCCCGAAGGG
AGAAAGGCGGACAGGTATCCGGTAAGCGGCAGGGTCGGAACAGGAGAGCGCACGAGG
GAGCTTCCAGGGGGAAACGCCTGGTATCTTTATAGTCCTGTCGGGTTTCGCCACCTCTG
ACTTGAGCGTCGATTTTTGTGATGCTCGTCAGGGGGGCGGAGCCTGTGGAAAAACGCC
AGCAACGCGGCCTTTTTACGGTTCCTGGCCTTTTGCTGGCCTTTTGCTCACATGTTCTTT
CCTGCGTTATCCCCTGATTCTGTGGATAACCGTATTACCGCCTTTGAGTGAGCTGATACC
GCTCGCCGCAGCCGAACGACCGAGCGCAGCGAGTCAGTGAGCGAGGAAGCCTGCATA
ACGCGAAGTAATCTTTTCGGTTTTAAAGAAAAAGGGCAGGGTGGTGACACCTTGCCCTT
TTTTGCCGGACTGCAGCGGCCGCTTCTAGaa

pSec-Reg linear version

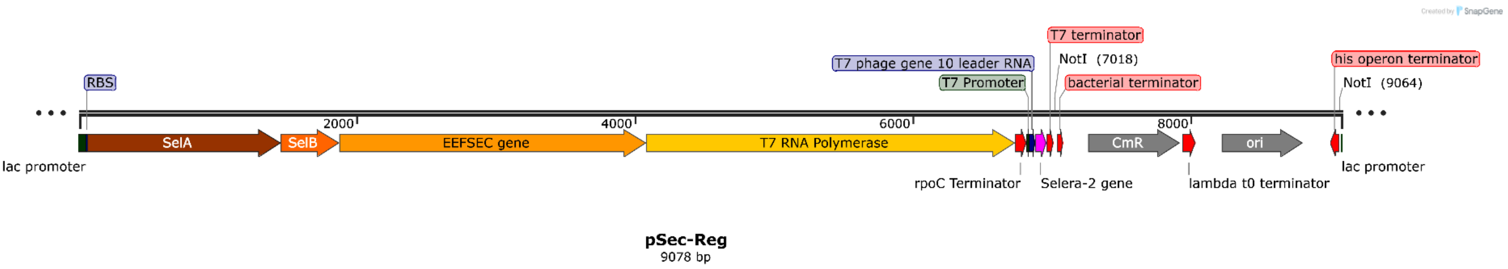

Virtual digest with Not I enzyme before Cassette and pSB1C3 assembly by cloning

pSB1C3 size after digestion: 2046 bp
Insert size after digestion: 7032 bp

Ladder	Life 1 kb Plus
1	pSB1C3 plasmid - NotI
2	Cassette - NotI

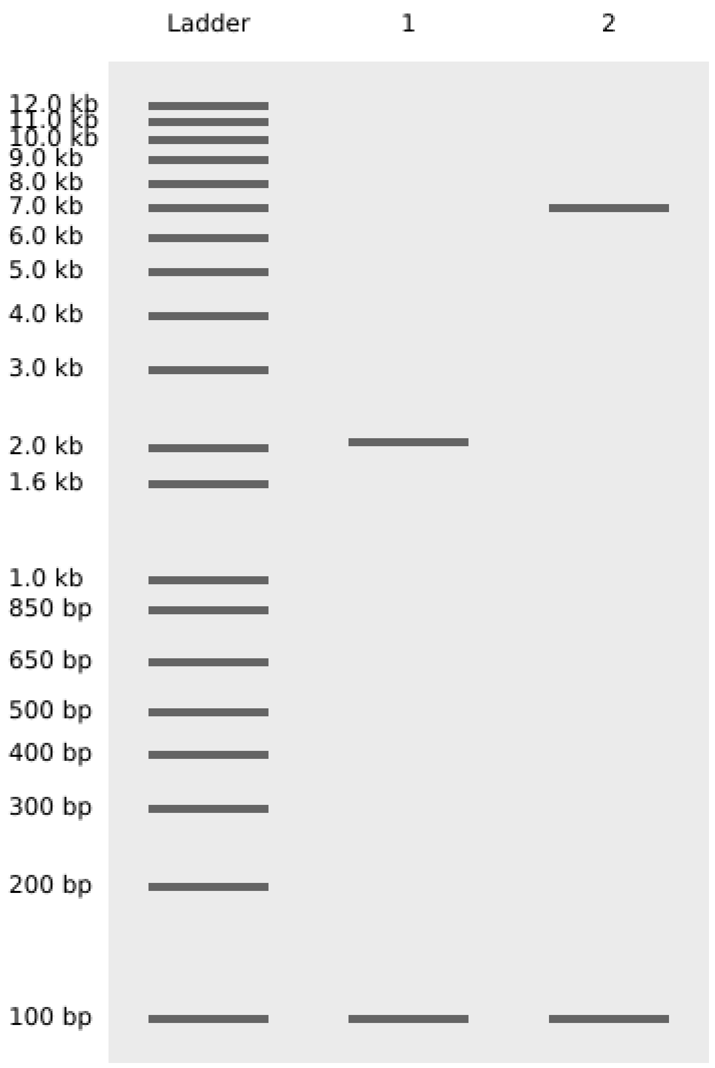

Supplement: Supplementary file 2 [file DataSheet1.pdf]
